# MultiPhen: Joint Model of Multiple Phenotypes Can Increase Discovery in GWAS

**DOI:** 10.1371/journal.pone.0034861

**Published:** 2012-05-02

**Authors:** Paul F. O’Reilly, Clive J. Hoggart, Yotsawat Pomyen, Federico C. F. Calboli, Paul Elliott, Marjo-Riitta Jarvelin, Lachlan J. M. Coin

**Affiliations:** 1 Department of Epidemiology and Biostatistics, Imperial College London, London, United Kingdom; 2 Department of Pedeatrics, Imperial College London, London, United Kingdom; 3 Department of Surgery and Cancer, Imperial College London, London, United Kingdom; 4 Translational Research Unit, Chulabhorn Research Institute, Bangkok, Thailand; 5 MRC-HPA Centre for Environment and Health, Imperial College London, London, United Kingdom; 6 Institute of Health Sciences, University of Oulu, Oulu, Finland; 7 National Institute of Health and Welfare, Oulu, Finland; 8 Department of Genomics of Common Disease, Imperial College London, London, United Kingdom; University of Hong Kong, Hong Kong

## Abstract

The genome-wide association study (GWAS) approach has discovered hundreds of genetic variants associated with diseases and quantitative traits. However, despite clinical overlap and statistical correlation between many phenotypes, GWAS are generally performed one-phenotype-at-a-time. Here we compare the performance of modelling multiple phenotypes jointly with that of the standard univariate approach. We introduce a new method and software, MultiPhen, that models multiple phenotypes simultaneously in a fast and interpretable way. By performing ordinal regression, MultiPhen tests the linear combination of phenotypes most associated with the genotypes at each SNP, and thus potentially captures effects hidden to single phenotype GWAS. We demonstrate via simulation that this approach provides a dramatic increase in power in many scenarios. There is a boost in power for variants that affect multiple phenotypes and for those that affect only one phenotype. While other multivariate methods have similar power gains, we describe several benefits of MultiPhen over these. In particular, we demonstrate that other multivariate methods that assume the genotypes are normally distributed, such as canonical correlation analysis (CCA) and MANOVA, can have highly inflated type-1 error rates when testing case-control or non-normal continuous phenotypes, while MultiPhen produces no such inflation. To test the performance of MultiPhen on real data we applied it to lipid traits in the Northern Finland Birth Cohort 1966 (NFBC1966). In these data MultiPhen discovers 21% more independent SNPs with known associations than the standard univariate GWAS approach, while applying MultiPhen in addition to the standard approach provides 37% increased discovery. The most associated linear combinations of the lipids estimated by MultiPhen at the leading SNPs accurately reflect the Friedewald Formula, suggesting that MultiPhen could be used to refine the definition of existing phenotypes or uncover novel heritable phenotypes.

## Introduction

Genome-wide association studies aim to identify associations between genotype and phenotype. While genotypes are well-defined biological entities, phenotypes are defined more subjectively and can relate to numerous biological processes. There has been much effort to characterise well-defined phenotypes that may correspond more to specific biological function for use in genome-wide association studies, but the definition of some phenotypes remains somewhat ad-hoc. Type 2 diabetes, the subject of the first major genome-wide association study [1], is diagnosed using a debated blood glucose threshold [2], the Metabolic Syndrome is commonly based on observing three of five criteria [3], while neuropsychiatric disorders rely on a complex range of overlapping clinical characteristics for diagnosis.

While most quantitative traits are less ambiguously defined, many are defined as mathematical functions of several measurements in an attempt to better capture the underlying biology than individual quantities. There have been many more genetic determinants of Body-mass-index (BMI), defined as the ratio of weight to height squared, discovered than those of weight [4], and the significance of rs8050136 in *FTO* was reported to be 11 orders of magnitude more significant for BMI than weight [5]. Illig et al. [6] investigated genetic association with metabolites and all metabolite ratios, using metabolite ratios as proxies for enzymatic reaction rates.

A primary aim of this study is to fully assess the performance of a multivariate approach to genome-wide association studies in comparison to that of the usual single-phenotype strategy. Rather than exploiting an available multivariate method, we introduce a new fast and interpretable approach, which addresses some of the limitations of previously suggested methods. In particular, we propose that linear combinations (equivalent to ratios on pre log-transformed phenotypes) of directly measured phenotypes may often capture unmeasured aspects of complex biological networks, such as reaction rates, protein mediators or other uncharacterised or clinically-inferred phenotypes. Modelling the association between linear combinations of phenotypes and the genotypes at each SNP may uncover genetic association hidden to both single phenotype GWAS and those based on *a priori* defining a phenotype as a fixed function of traits. Such an approach circumvents the problems of phenotype definition by enabling association of genotype with flexible linear combinations of robust measurements, and could potentially be exploited by biologists and clinicians to define phenotypes that better reflect underlying biological processes.

We introduce a new method, MultiPhen, that rapidly performs GWAS on multiple phenotypes by identifying the linear combination of the phenotypes most associated with genotype at each SNP. This is achieved by reversing the regression, such that genotype is regressed on phenotype, rather than phenotype-on-genotype as in the standard GWAS approach. By applying ordinal regression (proportional odds logistic regression), which models the genotype data as ordinal, multiple phenotypes can then be jointly modelled as predictors of the SNP genotypes to test for multi-phenotype associations (see Methods). This model makes no assumption on the phenotype distribution and so can accommodate both binary and continuous measurements. Our test for association is a likelihood ratio test for model fit, which provides a *P* value for evidence of association between the SNP and the phenotypes. The usual genome-wide significance level is applied. While several approaches to multivariate analysis of GWAS have been introduced in recent years, the univariate approach is still routinely favoured [7]–[9]. We believe that this is partly due to a lack of thorough comparison between a multivariate and the univariate approach, which we seek to address here, and also potentially due to limitations in the multivariate methods, such as: not allowing joint analysis of continuous and binary phenotypes [10]–[11], having inflated type 1 error [11], reducing the effective sample size by requiring cross-validation [12], and not explicitly modelling the correlation structure between traits [13]–[14]. MultiPhen does not suffer from these problems, but the benefit of using MultiPhen over ‘canonical correlation analysis’ (CCA) [15], when testing one-SNP-at-a-time, is less clear due to their similarity. MultiPhen and CCA (as implemented by Ferreira and Purcell [15]) both test the linear combination of the phenotypes most associated with the genotypes of a SNP against a null hypothesis of no association, but while CCA treats the genotypes as normally distributed MultiPhen appropriately models the genotypes as ordinal. Based on this, we specifically investigate whether MultiPhen offers better performance than CCA (which is equivalent to ‘reversed’ linear regression, with SNP as outcome, and MANOVA).

We show via simulation that MultiPhen has markedly higher power than the standard single-phenotype approach to detect SNP-phenotype associations in many scenarios, even for identifying variants that affect only one of the phenotypes, as a result of jointly modelling the phenotypes. In addition, there are several convenient aspects of adopting our approach. As the phenotypes are treated as predictors rather than outcomes, there is no need to ensure their normality via transformation. Our MultiPhen software (available as a CRAN package for R) appropriately handles imputed SNP data and CNVs, can optionally perform a score test, and can perform univariate linear (standard) and ordinal (reversed) regressions for comparison with multivariate results (see Methods). A further advantage of our approach is its computational speed; applying MultiPhen to 10 traits is around 1.3 times faster than the corresponding set of standard single-phenotype analyses. With a natural test for combining results across studies as the (weighted) sum of the likelihood ratios (or *z* scores) from each study, MultiPhen can be easily applied to perform meta-analysis of measured or imputed SNP data across a large number of phenotypes and studies. The similarity of the approach to that of the usual linear and logistic regressions performed in GWAS, and the presentation of *P* values for association in the usual way, make the approach highly interpretable and a natural extension of current methods for GWAS.

## Results

### Type 1 Error Rate Assessed by Simulation

We first assess whether MultiPhen, CCA [15] and the standard univariate approach have appropriate type 1 error by simulating sets of 100000 SNPs under the null hypothesis of no association, for 2 continuous phenotypes with correlations between the phenotypes of up to r = 0.9 (where r is Pearson’s correlation coefficient). Here and subsequently, the univariate *P* value is calculated as the minimum univariate *P* value across the tested phenotypes corrected for the effective number of tests by applying a “Nyholt-Šidák correction” [16] (see Methods). For normally distributed phenotypes tested at common and rare SNPs (MAF = 30% and 0.5%, N = 5000), MultiPhen, CCA and the Nyholt-Šidák corrected univariate approach generate uniform *P* values, and thus successfully control the false-positive rate (Figures S1 and S2). This is also the case when one of the phenotypes is simulated to have a distribution with outliers, such that the error term is simulated from a *t*-distribution with 3 degrees of freedom rather than a Normal distribution, when tested at common SNPs (Figure S3). However, there is substantial inflation of the statistics for both CCA and the univariate approach for low frequency variants when the phenotype distribution includes outliers (Figure S4). We also show that there is inflated type 1 error for CCA and the univariate approach in this scenario for common SNPs, with MAF = 5%, when the sample size is small, N = 200 (Figure S5). Finally, we investigate the type 1 error rates of the three methods when applied to binary phenotypes, and find that while all three methods have appropriate type 1 error rates when tested at SNPs with MAF = 30% (Figure S6) there is inflation of the statistics for CCA and the univariate approach when MAF = 0.5% and N = 5000 and when MAF = 5% and N = 200 (Figures S7 and S8). Tables S1, S2 and S3 provide the number of results with *P* values smaller than several thresholds in each of these scenarios, while Table 1 shows the number of results with *P*<1e^–5^, for which the expectation under the null is one, for each of the methods when the phenotypes have a correlation coefficient of 0.5. These results suggest that the assumption of normality of the genotypes made by the CCA method can critically compromise the approach in certain scenarios.

**Table 1:** Behaviour of the different methods under the null.

Phenotypes	MultiPhen			CCA			Univariate			
	30%	0.5%	5%, (N = 200)	30%	0.5%	5%, (N = 200)	30%	0.5%	5%, (N = 200)	
Continuous, no outliers	2	1	1	2	1	1	2	4	0	
Continuous with outliers	3	1	0	1	74	16	1	57	9	
Binary	2	2	1	1	6	17	0	8	22	

This table relates to the simulation study to test the type 1 error rates of MultiPhen, CCA, and the univariate approach, described in the text. The elements of the table show the number of results with *P*<1e^–5^ in the scenario described by the corresponding row and column (which give the minor allele frequencies) headers. Since 100000 replicates of SNP-phenotype associations were simulated under the null hypothesis of no association, the expectation for all elements of the table is 1; those with >1 indicating inflation of the type 1 error rate. Simulations with MAF = 30%, 0.5% were performed on a sample size of N = 5000. For the full results see Figures S1–S8 and Table S1–S3.

We note that when CCA is applied one-SNP-at-a-time, as in Ferreira and Purcell [15], its test statistic is equivalent to an *F*-test in a linear regression model with SNP genotypes as outcome and to a MANOVA test, and therefore these approaches have the same inflation of the type 1 error rate in the scenarios considered here.

### Statistical Power Assessed by Simulation

To formally compare the statistical power of MultiPhen, CCA and separately performed single phenotype analyses, we conducted a simulation study. We first investigate the potential power gains of using a multivariate approach over the standard univariate approach, then subsequently assess differences in power between the multivariate methods. We simulated replicates of a causal variant under several scenarios of its effect on two (simulated) continuous phenotypes. We simulated 10000 replicates of a causal SNP in a sample of 5000 individuals, where the risk allele explains 0.5% of the variance in the first phenotype and, in turn, 0.5%, 0.1% and 0% of the variance in the second phenotype. We simulate the variant as having different effects on the two ‘measured’ phenotypes in order to model association of the SNP with different linear combinations of the phenotypes. These scenarios were simulated across the range of between-phenotype correlations, from r = –0.9 to r = 0.9 in increments of 0.1. We first tested for SNP-phenotype associations by performing single phenotype analyses, calculating the Nyholt-Šidák corrected minimum *P* value from the two analyses for each SNP. Next we applied MultiPhen to the same data and performed the likelihood ratio test for overall model fit, obtaining a *P* value for association with the phenotypes (see Methods for details of simulation study).

**Figure 1:**
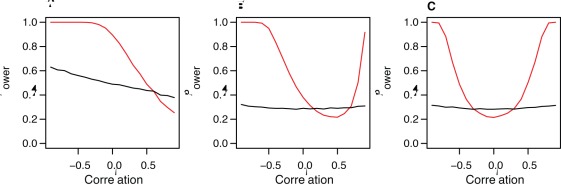
The power of MultiPhen in different scenarios of effect and correlation between phenotypes. Power results based on simulations described in the text for MultiPhen (red lines) and the standard single-phenotype approach (black lines). Left panel: causal variant explains 0.5% of phenotypic variance of both phenotypes. Middle panel: causal variant explains 0.5% on the phenotypic variance of the first phenotype and 0.1% of the variance in the second phenotype. Right panel: causal variant explains 0.5% of phenotypic variance of the first phenotype and 0% of the second phenotype.

**Figure 2:**
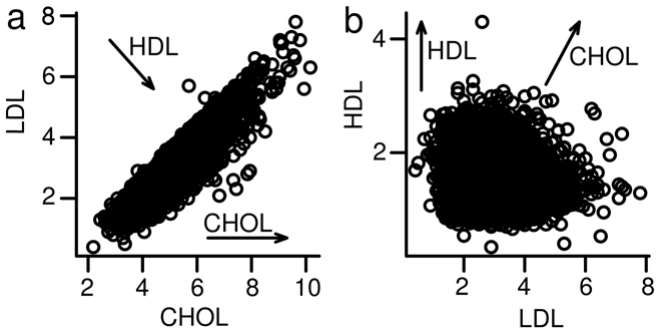
The correlation structure between pairs of lipids. The left panel shows the correlation structure between total cholesterol (CHOL) and low-density lipoprotein (LDL) in 5655 individuals from the Northern Finland Birth Cohort 1966. Each circle depicts the value of CHOL (X-axis) and LDL (Y-axis) in mmol/L for each individual. The right panel shows the correlation structure between low-density lipoprotein (LDL) and high-density lipoprotein (HDL), in mmol/L, in the same individuals. The arrows in each plot show the direction of effect of a variant affecting only CHOL or only HDL, such that the genotypes of individuals underlying each plotted point are more likely to contain risk alleles for the labelled lipid moving through the points in the direction of the arrow. The diagonal arrows are based on the Friedewald Formula (Friedewald.72). The arrows indicate that effects of variants can be in very different directions in the 2-dimensional spaces shown; the aim of modelling and testing linear combinations of phenotypes is to capture effects in any direction.

Figure 1 compares the power of the methods to identify associations at the genome-wide significance level (*P = *5x10^–8^) under these scenarios. MultiPhen outperforms the single-phenotype approach in detecting direct effects for the majority of the scenarios considered, and in many cases the boost in power is dramatic. MultiPhen performs particularly well when the genetic effects are not in the same direction as the correlation between the two phenotypes. For instance, when a variant only affects one of two highly correlated phenotypes, or a variant affects negatively correlated phenotypes in the same direction or positively correlated phenotypes in opposite directions (Figure S9). Exemplars of the last scenario are HDL variants, which may have opposite effects on total cholesterol and LDL (see Figure 2). If the genetic effects are consistent with the correlation between the two phenotypes, then the single phenotype approach is slightly more powerful, for example, if the variant has the same effect on two highly correlated phenotypes, or only affects one of two uncorrelated phenotypes.

We note that, from a theoretical perspective, the power of MultiPhen using two phenotypes P1 and P2, is worst when the association of P2 with genotype G is entirely explained by P1, that is when P2 is conditionally independent of G given P1. We show that this corresponds to the situation in which the ratio of the effect sizes β_2_/β_1_ = r (the correlation between the phenotypes), assuming P1 and P2 both have a variance of 1 (see Methods). This could occur if P2 is immediately downstream of P1 in a biological pathway with no other P2-regulators associated with G. Conversely MultiPhen is expected to perform better when β_2_/β_1_ is not equal to r, that is, when the genetic effects are not in the same direction as the correlation. While we would expect effects of variants that explain a large proportion of phenotypic variation to be consistent with the correlation of the phenotypes, this may not be the case for variants that explain only a small portion of phenotypic variation, such as those discovered in GWAS. Given the complexity of biological pathways, we suggest that in many cases genetic effects will not have the same correlation structure as the phenotypes themselves. In these circumstances, we would expect that MultiPhen has an advantage over traditional single-phenotype approaches.

To investigate the generalisability of the results of Figure 1 to more than two phenotypes we extended the simulations to three, five and ten phenotypes. In general MultiPhen performs even better with additional phenotypes in the scenarios where it outperforms the univariate approach, but even worse with more phenotypes when it has low power compared to the univariate approach (Figure S10). With a large number of correlated phenotypes the most likely scenario of effects may be that where the variant has different sized effects on several phenotypes, which is best captured by the middle panel of Figure S10 in which MultiPhen has improved overall performance with increasing phenotypes.

Next, we investigate the performance of MultiPhen when applied to case-control data. Figures S11 and S12 show that MultiPhen outperforms the univariate approach in the majority of the model space when applied to two case-control phenotypes, and when applied to a case-control phenotype and quantitative phenotype together.

Finally, we compared the statistical power of CCA (equivalently ‘reversed’ linear regression and MANOVA) and MultiPhen. Since the type 1 error rate of CCA is inflated for non-normal phenotypes in the scenarios we considered (see above), it is difficult to make a meaningful assessment of power in these scenarios. Therefore we restricted our analysis to normally distributed phenotypes. Figure S13 shows that while the power of CCA is in general marginally higher than MultiPhen, the difference is negligible. As a result, in the empirical example that follows we only compare MultiPhen with the standard univariate approach.

### Empirical Data Example: Lipid Traits in the NFBC1966

Next we describe the application of MultiPhen to lipid traits (total cholesterol, high and low density lipoprotein, and triglycerides: CHOL, HDL, LDL and TRIG) in the Northern Finland Birth Cohort 1966 (NFBC1966). Figures 2a and 2b illustrate the 2-dimensional correlation structure between CHOL and LDL, and LDL and HDL, respectively. We note from the Friedewald Formula [17] (in units of mmol/L),
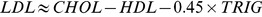
(1)that variants with an effect on CHOL will correspond to an effect in the direction of the X-axis in Figure 2a, as well is in the Y = X direction in Figure 2b; whereas variants with an effect on HDL correspond to an effect in the direction of the Y = –X direction in Figure 2a and in the direction of the Y-axis in Figure 2b. Thus, depending on which variables have been measured, the direction of the effect may not be along either one of the X or Y axes and may not be directly along any specific axis of interest. In fact we suggest that each casual variant may have an effect in a different direction. MultiPhen tests for variant effects on groups of correlated phenotypes without making a prior assumption about the direction of effect. While GWAS to date can be viewed as having tested for effects in a single direction in the n-dimensional space of n correlated phenotypes, we propose the use of MultiPhen to test for effects in any direction when data on multiple correlated phenotypes are available.

### Examining the Power of MultiPhen to Detect Lipid Associations

A study in 2010 [7] performed separate GWAS analyses on these four lipid traits (CHOL, LDL, HDL, TRIG) in over 100,000 individuals and discovered 95 independent SNPs associated with one or more of the traits. Here we exploit this extensive list of established associations to assess the performance of MultiPhen, using data from 4476 individuals in the Northern Finland Birth Cohort 1966 (NFBC1966). We performed single phenotype analyses and a MultiPhen analysis across the 95 SNPs (genotyped and imputed) to test for SNP-phenotype associations with the four lipids (see Methods). For the single phenotype analyses we selected the minimum *P* value from the analyses of each of the phenotypes for each SNP and adjusted it for multiple testing using a “Nyholt-Šidák correction” [16]. Table S4 shows the phenotype correlation-matrix for the 4 lipids.

Of the 95 known associated SNPs, 8 showed genome-wide significance using single phenotype analyses, while a total of 11 SNPs were genome-wide significant under either MultiPhen or the single phenotype analyses (Table 2). Furthermore, after excluding the most associated phenotype, MultiPhen still identified 7 SNPs as genome-wide significant, compared to only 2 significant single phenotype associations. The difference in *P* values in this case was almost uniformly in favour of MultiPhen, and for one SNP the *P* value was almost 15 orders of magnitude smaller, and approximately 5 orders of magnitude smaller for another 3 SNPs. Table 3 provides the regression coefficients underlying the MultiPhen models in the final column of Table 2. On average these linear combinations are similar to what we would expect from the Friedewald Formula (Equation 1). This suggests that MultiPhen is correctly identifying the linear combination of phenotypes that best approximates the most associated (unmeasured) phenotype. This highlights the potential utility in explicitly modelling combinations of phenotypes. MultiPhen also improves on the level of significance of the best univariate result when applied to all four lipids for 4 SNPs, indicating that these 4 SNPs may be associated with other lipid traits or phenotypes not considered here.

**Table 2:** Results under standard GWAS and MultiPhen approaches for genome-wide significant SNPs.

SNP	All 4 phenotypes				3 phenotypes after removing most associated			
	Best univariate		MultiPhen		Best univariate		MultiPhen	
	Trait	-log10 P¶	-log10 P	Diff.	Trait	-log10 P§	-log10 P	Diff.
rs3764261	HDL	**25.6**	**22.2**	–3.3	TRIG	1.3	**16**	**14.7**
rs4420638	LDL	**12.7**	**8.9**	–3.7	CHOL	**8.7**	**8.2**	–0.5
rs629301	LDL	**12.2**	**10.8**	–1.4	CHOL	**8.2**	**10.7**	2.5
rs964184	TRIG	**10.7**	**8.1**	–2.7	HDL	2.6	**7.4**	4.8
rs1367117	LDL	**9.2**	**7.3**	–1.9	CHOL	6.8	**7.8**	1
rs1532085	HDL	**8.8**	**9.3**	0.5	CHOL	1.6	4.4	2.8
rs6511720	LDL	**8.4**	5.6	–2.7	CHOL	6.2	5.4	–0.9
rs1260326	TRIG	**7.8**	5.4	–2.3	CHOL	1	2.5	1.5
rs1042034	LDL	6.7	**9.6**	2.9	TRIG	5.1	**10**	4.9
rs12678919	TRIG	6.2	**7.8**	1.5	HDL	3.9	5	1.2
rs174546	LDL	4.9	**8.5**	3.7	CHOL	3.3	**8.9**	5.6

¶ Nyholt-Šidák corrected for 4 comparisons. § Nyholt-Šidák corrected for 3 comparisons. Results compare univariate and MultiPhen *P* values, presented on the -log10 scale for ease of comparison, for all SNPs with genome-wide significant *P* values (>7.301 on the -log10 scale) from either approach. Genome-wide significant results shown in bold. The difference in terms of orders of magnitude of the MultiPhen *P* value on all phenotypes is relative to the most associated univariate phenotype; and the order of magnitude difference for MultiPhen where the most associated phenotype is excluded is relative to the univariate result also excluding the most associated phenotype.

**Table 3:** Most associated linear combinations of phenotypes at genome-wide significant SNPs.

SNP	Most associated trait	Maximally associated linear combination after removing most associated trait			
		CHOL	LDL	HDL	TRIG
rs3764261	HDL	1	−0.97	–	−0.37
rs4420638	LDL	1	–	−1.59	−0.75
rs629301	LDL	1	–	−1.07	−0.74
rs964184	TRIG	1	−0.87	−1.18	–
rs1367117	LDL	1	–	−1.38	−0.71
rs1532085	HDL	1	−0.91	–	−0.11
rs6511720	LDL	1	–	−0.78	−0.31
rs1260326	TRIG	1	−0.94	−1.14	–
rs1042034	LDL	1	–	−3.3	0.47
rs12678919	TRIG	1	−1.19	−1.93	–
rs174546	LDL	1	–	−0.51	−1.45
**Average (Median)**		**1**	**−0.94**	**−1.18**	**−0.54**
**Friedewald expected**		**1**	**−1**	**−1**	**−0.45**

&ast; indicates that the SNP did not have a univariate genome-wide significant *P* value. Each row indicates the linear combination of phenotypes (given by the corresponding regression coefficients) which is most associated with the given SNP under the MultiPhen regression, after removing the most associated phenotype. The regression coefficients have been scaled so that the CHOL coefficient is always equal to one. The last row contains the expected coefficients according to the Friedewald Formula (Equation 1).

**Figure 3:**
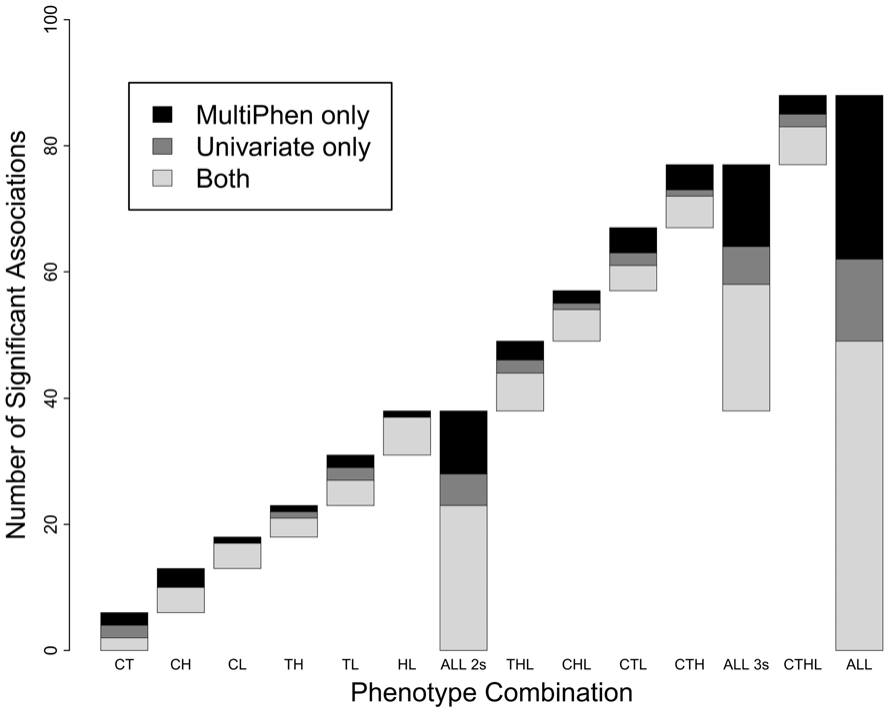
Genome-wide significant results from standard GWAS approach and MultiPhen tested on combinations of the lipids using NFBC1966 data. Each bar shows the number of SNPs reaching genome-wide significance for a given phenotype-combination analysis (specified by the first letters of each trait, such that CHL refers to an analysis on the CHOL, HDL and LDL), with the SNPs discovered by both the univariate approach and MultiPhen shown by the white segment of the bar, the SNPs discovered by the univariate approach only shown by the grey segment, and the SNPs discovered by MultiPhen only illustrated by the black segment. The bars labelled ALL2 and ALL3 combine results across analyses on all combinations of two and three lipid traits, respectively, while ALL combines the results across the analyses of all 2, 3 and 4 combinations of the traits. A complete breakdown of these results is presented in Tables S5, S6, S7, S8, S9, S10, S11, S12, S13, S14, S15.

We also assessed the performance of MultiPhen relative to the univariate approach over different subsets of our measured lipid phenotypes, each reflecting hypothetical studies with measurements available only on certain traits, in order to gain further insight into the relative performance of the two approaches. We analysed all 3-phenotype combinations of the lipids (4 analyses) and all 2-phenotype combinations (6 analyses) and compared the number of genome-wide significant SNPs between MultiPhen and the univariate analyses (Figure 3 and Tables S5, S6, S7, S8, S9, S10, S11, S12, S13, S14, S15). Of the 11 analyses performed in total, MultiPhen produced a larger number of genome-wide significant SNPs than the univariate approach in 8 analyses and the same number in 3. While in some examples, the single phenotype analysis generates *P* values 2–3 orders of magnitude smaller than MultiPhen, no univariate SNP-phenotype association *P* values are 4 orders of magnitude or more smaller than the corresponding MultiPhen result. In contrast, there are 10 MultiPhen *P* values smaller than the univariate *P* values by at least 4 orders. MultiPhen identifies 21% more SNP-phenotype associations than the univariate approach, with 75 compared to 62 significant associations, relating to 12 SNPs. By combining all significant univariate and MultiPhen SNPs there are a total of 85 SNP-phenotype associations with *P*<2.5x10^–8^ (conservative Bonferonni correction applied), compared to 62 using the univariate strategy only. Therefore, based on these empirical findings, MultiPhen provides a 21% increase in detection of susceptibility loci if used instead of the single phenotype approach, and a 37% increase in detection if used in addition to univariate analyses.

In order to test for false-positive results and assess any inflation of the test statistic, we applied MultiPhen to the four lipids genome-wide. The only genome-wide significant findings were from loci harbouring one of the established 95 lipid SNPs, confirming no false-positive results in these analyses. The inflation factor of the test statistic calculated from the genome-wide MultiPhen results was 1.06, indicating no unusual inflation of the test statistic. While we did not detect novel susceptibility loci using MultiPhen in the NFBC1966, this was not unexpected given that the sample size of Teslovich et al. [7] that reported the 95 SNPs was ∼21 times greater than the present study.

## Discussion

Since the emergence of GWAS in 2007 with two seminal publications [1], [18], the approach of testing the association between a genetic variant and phenotype, each one-at-time, has remained the method of choice. However, despite the development of multivariate methods, so far there has been limited application to GWAS datasets. In this report, we have shown with both real and extensively simulated data the extent of the power gains that can be achieved through the multivariate approach.

We have introduced a new multivariate method, MultiPhen, which addresses limitations of alternative multivariate methods. Key advantages of our approach are its computational speed, the modelling and subsequent availability of the linear combination of phenotypes most associated with each genotype, and its application to both quantitative – regardless of phenotype distribution – and case-control data.

We demonstrated that the MultiPhen model provides appropriate type 1 error rate when applied to both common and rare variants, non-normal continuous phenotypes and binary phenotypes. In contrast, CCA [15] and the univariate approach used here have inflated type 1 error rates when applied to non-normal continuous phenotypes or binary phenotypes at low frequency variants. We also demonstrated via application to simulated and real data that MultiPhen can achieve substantial increases in statistical power to detect true associations. Simulations show that while power is dependent on the correlation structure of the phenotypes and the genetic effects on them, and can be marginally greater for the univariate approach, most of the model space corresponds to significantly higher power for MultiPhen. We find that CCA and MultiPhen have almost the same statistical power when applied to normally distributed phenotypes. The dramatic gains in power for MultiPhen over the univariate approach when the genetic effects and phenotypic correlations are discordant, suggest that MultiPhen may discover causal variants not amenable to discovery by the univariate approach even with large increases in sample size. The application of MultiPhen to lipid traits in the NFBC1966 provides supportive evidence for the performance of MultiPhen using real data.

By considering all 2, 3, and 4 phenotype combinations of the 4 lipid traits we were able to test empirically the performance of MultiPhen applied to phenotypes with different correlation structures, in comparison with the univariate approach. These results indicate a 21% greater yield of SNP-phenotype associations when MultiPhen is applied instead of the univariate approach, and a 37% increased yield over the univariate analysis when significant univariate and MultiPhen results are combined. However, given that these analyses relate to a limited number of phenotypes and significant SNPs, these figures should not be considered accurate estimates of the general performance of MultiPhen over the univariate approach, which can only be obtained from extensive future use on a variety of phenotypes. Our findings on the lipid data are, however, supportive of the results from our simulation study in indicating that MultiPhen may lead to greater discovery when used instead of the univariate approach, and suggest that applying both approaches and combining results could lead to even greater discovery.

We consider it likely that causal variants commonly influence many separately defined phenotypes [19], with protein products acting as intermediaries in complex causal networks. We suggest that this most likely applies to correlated phenotypes since these must share risk factors and most likely have common biological pathways. We propose that the association between genotype and an unmeasured protein product, or other unmeasured mediator or uncharacterised phenotype, may be captured by a linear combination of measured phenotypes that are affected. Moreover, we have shown that we can recapitulate the Friedewald Formula (Equation 1) for the 4 lipid traits studied here using the average regression coefficient over multiple SNPs. This strategy may help to refine the definition of existing phenotypes and also suggest novel phenotypes for further investigation, thus providing insights into underlying biological processes and diseases.

An overlap in loci identified by GWAS on different phenotypes [20], as well as greater availability of data on multiple phenotypes in GWAS consortia, has led to increased interest in studying multiple phenotypes together in GWAS [21]. Here we have introduced a simple and computationally fast method and software for performing GWAS on multiple phenotypes jointly, suitable for application to directly genotyped or imputed SNP or CNV data for association testing with quantitative or case-control phenotypes. MultiPhen should be considered a discovery tool for application to multiple correlated phenotypes that makes no prior assumptions about the nature of the genetic effects on the phenotypes. Over a wide range of plausible scenarios, MultiPhen can achieve marked increases in power, both when the genetic variant affects more than one phenotype and when it affects only a single phenotype (Figure 1). We propose the use of MultiPhen in future GWAS on multiple correlated phenotypes as a rapid, user-friendly and effective means to reveal novel susceptibility loci that would have been missed by the standard single-phenotype approach.

## Materials and Methods

### Ethics Statement

This study was approved by the Ethical Committee of the Northern Ostrobothnia Hospital District; written, informed consent was obtained from all participants.

### MultiPhen Approach

In the standard GWAS approach, when considering a quantitative phenotype, a linear regression is usually performed of phenotype, *Y*, on genotype, *X*. We let *Y_i_* = {*Y_i1_*, …, *Y_iK_*} denote the phenotype data corresponding to *K* phenotypes for an individual *i* and *X_i_* = {*X_i1_*, …, *X_iG_*} denote their genotype data at *G* SNPs, where *X_ig_ ε* {0,1,2}. The regression performed at a SNP, *g*, and a phenotype, *k*, to test for association between the SNP genotypes and the phenotype is thus:
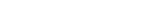
where *ε_igk_* is the residual error assumed to be normally distributed. The null hypothesis of no association between SNP and genotype can be tested by performing a t-test on the null hypothesis 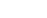.

In our MultiPhen approach we invert the regression so that the SNP genotypes, *X*, become the dependent variable, and the *K* phenotypes under study become the predictor variables. The genotype data is an allele count and is therefore modelled using ordinal regression; we use proportional odds logistic regression. This model defines the class probabilities as follows.
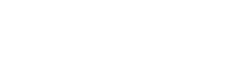



At each SNP *g = 1,…,G w*e use a likelihood ratio test to test the null hypothesis *β_g1_ = … = β_gK_ = *0. This test does not assume Hardy-Weinberg Equilibrium.

### Application to Lipid Trait Data in the NFBC1966

Complete genotype and lipid trait data were available on 4476 individuals (5655 individuals with complete lipid data, used in Figure 2) from the Northern Finland Birth Cohort 1966 (NFBC1966) across the 95 lipid SNPs established in Teshlovich et al. [7]. In the NFBC1966, mothers living in the two northern-most provinces of Finland were invited to participate if they had expected delivery dates during 1966. At age 31, 5,923 individuals from the cohort still living in the Helsinki area or Northern Finland were asked to participate in a detailed biological and medical examination, from which the genotype and lipid data derive. Genotypes that were not directly measured were imputed using IMPUTE [22]. Single phenotype analyses were performed using linear regression in the MultiPhen package (see below), and the *P* values obtained from each analysis at each SNP were subsequently corrected for multiple testing using a “Nyholt- Šidák correction” [16] based on the correlation matrix of the lipids (Table S4); where the number of effective tests is calculated using the approach taken in Nyholt 2004 [16] and then used to compute a Šidák corrected *P* value [23].

### Simulation Study

We tested the MultiPhen model using simulated genotype and phenotype data. Genotype data was simulated for 5000 individuals assuming a minor allele frequency of 20% and Hardy-Weinberg Equilibrium. For each individual two continuous phenotypes were simulated given the genotype data, controlling the heritability of each phenotype and the correlation between phenotypes. The two phenotypes were simulated from the following models:
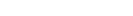


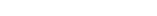
where *X_i_* is the simulated genotype data taking values 0, 1 and 2, *Ei* ∼ *N(0,1)* represents a common environmental effect affecting both phenotypes, and ε is a random effect distributed *N(0,σ^2^)*. Heritability is defined as the proportion of the variance of the trait due to genetic heterogeneity. According to the above models the variances of the two traits are given by:



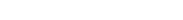


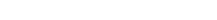
where *p* is the allele frequency. Therefore, if we choose parameters such that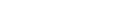, then the heritability of trait *k* is 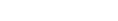.

If the traits are centered to both have mean 0, then the correlation between the traits is:
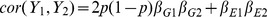
(2)


To simulate all scenarios in the simulation study we solve for 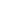,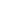 and *σ^2^* conditional on 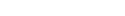 and the desired correlation (2).

MultiPhen has its worst power when the association of *Y_2_* with *X* is explained by Y_1_ (or vice versa). That is to say, when the residuals of *Y_2_*, regressing out the effect of *Y_1_*, are independent of *X*. If 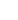 are the residuals of *Y_2_* regressed on *Y_1_* for individuals *i = 1, …, N*, then:
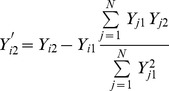
(3)


Regressing *Y_1_* and *Y_2_* on *X* gives:
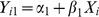
(4)

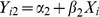
(5)


Substituting (4) and (5) into (3) gives:
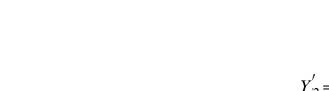
Therefore 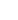 is independent of *X_i_* when 
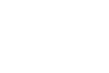


For standardised phenotype data, 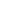
is independent of *X_i_* when 
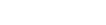
, that is, when the ratio of the genetic effects is equal to the correlation between the phenotypes.

Binary traits were simulated assuming a classical liability threshold model. We used the same simulation as for continuous traits assuming the continuous traits were liability phenotypes. In this model all individuals with liability phenotypes greater than a threshold *t* were assumed to be a case for that trait. Heritability on the observed scale is given by:
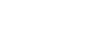



Where *q* is the disease prevalence and *z* is the height of the normal pdf at the threshold value *t*. All correlations on Figures relating to simulations involving binary traits (Figures S11 and S12) are with respect to liabilities. Case-control data was simulated by randomly sampling cases and controls from a large simulated population.

### MultiPhen Software

The MultiPhen software was written in the R statistical language. R users can download and install MultiPhen from CRAN (http://cran.r-project.org/) or any CRAN mirror, with documentation available here:

http://cran.r-project.org/web/packages/MultiPhen/MultiPhen.pdf. Alternatively, a Java executable can be downloaded from http://www1.imperial.ac.uk/medicine/people/l.coin/.

## Supporting Information

Figure S1 **Behaviour under the null: without outliers, MAF = 30%.** QQ-plots, corresponding to MultiPhen, CCA and the univariate approach (Nyolt-Šidák corrected) applied to data simulated under the null hypothesis of no SNP-phenotype effects (100000 replicates for each), sample size N = 5000, with MAF of simulated SNPs of 30% and 2 normally distributed phenotypes (without outliers) and correlations between the phenotypes of 0, 0.5 and 0.9. The plots show that all three methods have an appropriate null distribution, even when the correlation between the phenotypes is high.
(TIFF)

Figure S2 **Behaviour under the null: without outliers, MAF = 0.5%.** QQ-plots, corresponding to MultiPhen, CCA and the univariate approach (Nyolt-Šidák corrected) applied to data simulated under the null hypothesis of no SNP-phenotype effects (100000 replicates for each), sample size N = 5000, with MAF of simulated SNPs of 0.5% and 2 normally distributed phenotypes (without outliers) and correlations between the phenotypes of 0, 0.5 and 0.9. The plots show that all three methods have an appropriate null distribution, even when the correlation between the phenotypes is high.
(TIFF)

Figure S3 **Behaviour under the null: with outliers, MAF = 30%.** QQ-plots, corresponding to MultiPhen, CCA and the univariate approach (Nyolt-Šidák corrected) applied to data simulated under the null hypothesis of no SNP-phenotype effects (100000 replicates for each), sample size N = 5000, with MAF of simulated SNPs of 30% and 2 phenotypes where one has an outlier distribution and correlations between the phenotypes of 0, 0.5 and 0.9. The plots show that all three methods have an appropriate null distribution, even when the correlation between the phenotypes is high.
(TIFF)

Figure S4 **Behaviour under the null: with outliers, MAF = 0.5%.** QQ-plots, corresponding to MultiPhen, CCA and the univariate approach (Nyolt-Šidák corrected) applied to data simulated under the null hypothesis of no SNP-phenotype effects (100000 replicates for each), sample size N = 5000, with MAF of simulated SNPs of 0.5% and 2 phenotypes where one has an outlier distribution and correlations between the phenotypes of 0, 0.5 and 0.9. The plots show that while MultiPhen has an appropriate null distribution, even when the correlation between the phenotypes is high, the other two approaches have a highly inflated null distribution, which would produce an extremely high type 1 error rate, irrespective of the correlation between the phenotypes.
(TIFF)

Figure S5 **Behaviour under the null: with outliers, MAF = 5% (N = 200).** QQ-plots, corresponding to MultiPhen, CCA and the univariate approach (Nyolt-Šidák corrected) applied to data simulated under the null hypothesis of no SNP-phenotype effects (100000 replicates for each), sample size N = 200, with MAF of simulated SNPs of 5% and 2 phenotypes where one has an outlier distribution and correlations between the phenotypes of 0, 0.5 and 0.9. The plots show that while MultiPhen has an appropriate null distribution, even when the correlation between the phenotypes is high, the other two approaches have an inflated null distribution. Thus CCA and the univariate approach can have a high type 1 error rate for common, as well as rare, variants.
(TIFF)

Figure S6 **Behaviour under the null: binary phenotypes, MAF = 30%.** QQ-plots, corresponding to MultiPhen, CCA and the univariate approach (Nyolt-Šidák corrected) applied to case-control study data simulated under the null hypothesis of no SNP-phenotype effects (100000 replicates for each), with sample size N = 5000 such that the first phenotype has 50% cases and controls whereas the second phenotype has 10% cases and 90% controls (case-control status defined according to the simulated values of underlying normally distributed continuous phenotypes). The MAF of the simulated SNPs is 30% and the correlations between the phenotypes (on a liability scale) are 0, 0.5 and 0.9. The plots show that all three methods have an appropriate null distribution when there are no SNP-phenotype effects, even when correlation between the phenotypes is high.
(TIFF)

Figure S7 **Behaviour under the null: binary phenotypes, MAF = 0.5%.** QQ-plots, corresponding to MultiPhen, CCA and the univariate approach (Nyolt-Šidák corrected) applied to case-control study data simulated under the null hypothesis of no SNP-phenotype effects (100000 replicates for each), with sample size N = 5000 such that the first phenotype has 50% cases and controls whereas the second phenotype has 10% cases and 90% controls (case-control status defined according to the simulated values of underlying normally distributed continuous phenotypes). The MAF of the simulated SNPs is 0.5% and the correlations between the phenotypes (on a liability scale) are 0, 0.5 and 0.9. The plots show that while MultiPhen has an appropriate null distribution, even when the correlation between the phenotypes is high, the other two approaches have an inflated null distribution.
(TIFF)

Figure S8 **Behaviour under the null: binary phenotypes, MAF = 5% (N = 200).** QQ-plots, corresponding to MultiPhen, CCA and the univariate approach (Nyolt-Šidák corrected) applied to case-control study data simulated under the null hypothesis of no SNP-phenotype effects (100000 replicates for each), with sample size N = 200 such that the first phenotype has 50% cases and controls whereas the second phenotype has 10% cases and 90% controls (case-control status defined according to the simulated values of underlying normally distributed continuous phenotypes). The MAF of the simulated SNPs is 5% and the correlations between the phenotypes (on a liability scale) are 0, 0.5 and 0.9. The plots show that while MultiPhen has an appropriate null distribution, even when the correlation between the phenotypes is high, the other two approaches have an inflated null distribution.
(TIFF)

Figure S9 **The power of MultiPhen in scenarios where the effect on the phenotypes is in the opposite direction.** Power results based on simulations described in the text for MultiPhen (red lines) and the standard single-phenotype approach (black lines). Left panel: causal variant explains 0.5% of phenotypic variance of both phenotypes but in opposite directions. Right panel: causal variant explains 0.5% of the phenotypic variance of the first phenotype and 0.1% of the variance in the second phenotype in the opposite direction of the effect on the first. The X-axis corresponds to the correlation (Pearson’s correlation coefficient, r) between the phenotypes.
(TIFF)

Figure S10 **The power of MultiPhen applied to two, three, five and ten phenotypes.** Power results based on simulations described in the text for MultiPhen (red lines) and the standard single-phenotype approach (black lines), for 2 (bold), 3 (dashed), 5 (dotted), and 10 (dashed/dotted) phenotypes. Left panel: causal variant explains 0.5% of phenotypic variance of each phenotype. Middle panel: causal variant explains 0.5% of the phenotypic variance of the first phenotype and 0.1% of the variance in each of the other phenotypes. Right panel: causal variant explains 0.5% of phenotypic variance of the first phenotype and 0% of each of the other phenotypes. The X-axis corresponds to the correlation (Pearson’s correlation coefficient, r) between all pairs of phenotypes (that is, the correlation between each pair of phenotypes is the same at each point of the X-axis). The results are truncated when the pairwise correlations are low for multiple phenotypes because the corresponding correlation matrices of the phenotypes are not positive definite and so the multivariate gaussian distributions cannot be sampled via the Cholesky decomposition that we perform.
(TIFF)

Figure S11 **The power of MultiPhen applied to two case-control phenotypes.** Power results based on simulations described in the text for MultiPhen (red lines) and the standard single-phenotype approach (black lines) applied to two independent case-control studies, each with 1000 cases and 1000 controls where case-control status is used as the predictor variables. Heritability on the liability scale is 0.1% for one phenotype and 0.1% (left panel), 0.05% (middle panel) and 0% (right panel) for the other phenotype. Solid and dashed are for disease prevalences of 1% and 0.5%, respectively, for both studies. Correlation on the X-axis is with respect to the liability scale.
(TIFF)

Figure S12 **The power of MultiPhen applied to one case-control and one quantitative phenotype.** The power of MultiPhen applied to a case-control study with 1000 cases and 1000 controls using case-control status and a single measured quantitative phenotype as predictor variables. Heritability of the continuous phenotype is 0.2%; heritability of the binary trait on the liability scale is: 0.5% (a), 0.1% (b) and 0% (c). Solid and dashed lines are for disease prevalence of 1% and 0.5% respectively. Correlation on the X-axis is with respect to the liability scale.
(TIFF)

Figure S13 **The power of CCA, compared to MultiPhen and the univariate approach.** Power results based on simulations described in the text for CCA (blue), MultiPhen (red) and the single-phenotype approach (black), when applied to two continuous normally distributed phenotypes where the causal variant explains 0.5% of the phenotypic variance of the first phenotype and 0.1% of the variance of the other phenotype, with the simulated SNPs having MAF = 30%. The X-axis corresponds to the correlation (Pearson’s correlation coefficient, r) between the phenotypes. The difference in statistical power between CCA and MultiPhen in this scenario is reflective of the difference in power when the effect of the causal variants on the phenotypes differs and for different allele frequencies of the causal variant (data not shown).
(TIFF)

Table S1 **Behaviour under the null: r = 0.** The table relates to the simulation study to test the behaviour of the different methods under the null hypothesis of no association, described in the text and presented in Figures S1–S8. The elements of the table correspond to the number of results with *P* values smaller than that of the corresponding column header (expected number given in the second row of the column header), with the simulation scenario given in the left-hand column. This table gives the results for all simulations where the two phenotypes have a correlation of r = 0.
(PDF)

Table S2 **Behaviour under the null: r = 0.5.** The table relates to the simulation study to test the behaviour of the different methods under the null hypothesis of no association, described in the text and presented in Figures S1–S8. The elements of the table correspond to the number of results with *P* values smaller than that of the corresponding column header (expected number given in the second row of the column header), with the simulation scenario given in the left-hand column. This table gives the results for all simulations where the two phenotypes have a correlation of r = 0.5.
(PDF)

Table S3 **Behaviour under the null: r = 0.9.** The table relates to the simulation study to test the behaviour of the different methods under the null hypothesis of no association, described in the text and presented in Figures S1–S8. The elements of the table correspond to the number of results with *P* values smaller than that of the corresponding column header (expected number given in the second row of the column header), with the simulation scenario given in the left-hand column. This table gives the results for all simulations where the two phenotypes have a correlation of r = 0.9.
(PDF)

Table S4 **Correlation matrix for the 4 lipids (CHOL, TRIG, HDL, LDL) based on the NFBC1966 data.** The upper triangular elements of the correlation matrix show the pairwise Pearson’s correlation coefficient (r) between each pair of the traits: total cholesterol (CHOL), triglycerides (TRIG), high-density lipoprotein (HDL) and low-density lipoprotein (LDL).
(PDF)

Table S5 **Results under standard GWAS and MultiPhen approaches for genome-wide significant SNPs: CHOL-TRIG-HDL-LDL combination.** Results compare univariate and MultiPhen *P* values, presented on the -log10 scale for ease of comparison, for all SNPs with genome-wide significant *P* values (>7.301 on the -log10 scale) from either approach. Genome-wide significant results shown in bold (only the smallest univariate result highlighted since this corresponds to the *P* value for the group of single phenotype analyses. Note, all univariate results are Nyholt-Šidák corrected). The difference in terms of orders of magnitude of the MultiPhen *P* value and the smallest univariate *P* value for each SNP is given in the final column.
(PDF)

Table S6 **Results under standard GWAS and MultiPhen approaches for genome-wide significant SNPs: TRIG-HDL-LDL combination.** Results compare univariate and MultiPhen *P* values, presented on the -log10 scale for ease of comparison, for all SNPs with genome-wide significant *P* values (>7.301 on the -log10 scale) from either approach. Genome-wide significant results shown in bold (only the smallest univariate result highlighted since this corresponds to the *P* value for the group of single phenotype analyses. Note, all univariate results are Nyholt-Šidák corrected). The difference in terms of orders of magnitude of the MultiPhen *P* value and the smallest univariate *P* value for each SNP is given in the final column.
(PDF)

Table S7 **Results under standard GWAS and MultiPhen approaches for genome-wide significant SNPs: CHOL-HDL-LDL combination.** Results compare univariate and MultiPhen *P* values, presented on the -log10 scale for ease of comparison, for all SNPs with genome-wide significant *P* values (>7.301 on the -log10 scale) from either approach. Genome-wide significant results shown in bold (only the smallest univariate result highlighted since this corresponds to the *P* value for the group of single phenotype analyses. Note, all univariate results are Nyholt-Šidák corrected). The difference in terms of orders of magnitude of the MultiPhen *P* value and the smallest univariate *P* value for each SNP is given in the final column.
(PDF)

Table S8 **Results under standard GWAS and MultiPhen approaches for genome-wide significant SNPs: CHOL-TRIG-LDL combination.** Results compare univariate and MultiPhen *P* values, presented on the -log10 scale for ease of comparison, for all SNPs with genome-wide significant *P* values (>7.301 on the -log10 scale) from either approach. Genome-wide significant results shown in bold (only the smallest univariate result highlighted since this corresponds to the *P* value for the group of single phenotype analyses. Note, all univariate results are Nyholt-Šidák corrected). The difference in terms of orders of magnitude of the MultiPhen *P* value and the smallest univariate *P* value for each SNP is given in the final column.
(PDF)

Table S9 **Results under standard GWAS and MultiPhen approaches for genome-wide significant SNPs: CHOL-TRIG-HDL combination.** Results compare univariate and MultiPhen *P* values, presented on the -log10 scale for ease of comparison, for all SNPs with genome-wide significant *P* values (>7.301 on the -log10 scale) from either approach. Genome-wide significant results shown in bold (only the smallest univariate result highlighted since this corresponds to the *P* value for the group of single phenotype analyses. Note, all univariate results are Nyholt-Šidák corrected). The difference in terms of orders of magnitude of the MultiPhen *P* value and the smallest univariate *P* value for each SNP is given in the final column.
(PDF)

Table S10 **Results under standard GWAS and MultiPhen approaches for genome-wide significant SNPs: CHOL-TRIG combination.** Results compare univariate and MultiPhen *P* values, presented on the -log10 scale for ease of comparison, for all SNPs with genome-wide significant *P* values (>7.301 on the -log10 scale) from either approach. Genome-wide significant results shown in bold (only the smallest univariate result highlighted since this corresponds to the *P* value for the group of single phenotype analyses. Note, all univariate results are Nyholt-Šidák corrected). The difference in terms of orders of magnitude of the MultiPhen *P* value and the smallest univariate *P* value for each SNP is given in the final column.
(PDF)

Table S11 **Results under standard GWAS and MultiPhen approaches for genome-wide significant SNPs: CHOL-HDL combination.** Results compare univariate and MultiPhen *P* values, presented on the -log10 scale for ease of comparison, for all SNPs with genome-wide significant *P* values (>7.301 on the -log10 scale) from either approach. Genome-wide significant results shown in bold (only the smallest univariate result highlighted since this corresponds to the *P* value for the group of single phenotype analyses. Note, all univariate results are Nyholt-Šidák corrected). The difference in terms of orders of magnitude of the MultiPhen *P* value and the smallest univariate *P* value for each SNP is given in the final column.
(PDF)

Table S12 **Results under standard GWAS and MultiPhen approaches for genome-wide significant SNPs: CHOL-LDL combination.** Results compare univariate and MultiPhen *P* values, presented on the -log10 scale for ease of comparison, for all SNPs with genome-wide significant *P* values (>7.301 on the -log10 scale) from either approach. Genome-wide significant results shown in bold (only the smallest univariate result highlighted since this corresponds to the *P* value for the group of single phenotype analyses. Note, all univariate results are Nyholt-Šidák corrected). The difference in terms of orders of magnitude of the MultiPhen *P* value and the smallest univariate *P* value for each SNP is given in the final column.
(PDF)

Table S13 **Results under standard GWAS and MultiPhen approaches for genome-wide significant SNPs: TRIG-HDL combination.** Results compare univariate and MultiPhen *P* values, presented on the -log10 scale for ease of comparison, for all SNPs with genome-wide significant *P* values (>7.301 on the -log10 scale) from either approach. Genome-wide significant results shown in bold (only the smallest univariate result highlighted since this corresponds to the *P* value for the group of single phenotype analyses. Note, all univariate results are Nyholt-Šidák corrected). The difference in terms of orders of magnitude of the MultiPhen *P* value and the smallest univariate *P* value for each SNP is given in the final column.
(PDF)

Table S14 **Results under standard GWAS and MultiPhen approaches for genome-wide significant SNPs: TRIG-LDL combination.** Results compare univariate and MultiPhen *P* values, presented on the -log10 scale for ease of comparison, for all SNPs with genome-wide significant *P* values (>7.301 on the -log10 scale) from either approach. Genome-wide significant results shown in bold (only the smallest univariate result highlighted since this corresponds to the *P* value for the group of single phenotype analyses. Note, all univariate results are Nyholt-Šidák corrected). The difference in terms of orders of magnitude of the MultiPhen *P* value and the smallest univariate *P* value for each SNP is given in the final column.
(PDF)

Table S15 **Results under standard GWAS and MultiPhen approaches for genome-wide significant SNPs: HDL-LDL combination.** Results compare univariate and MultiPhen *P* values, presented on the -log10 scale for ease of comparison, for all SNPs with genome-wide significant *P* values (>7.301 on the -log10 scale) from either approach. Genome-wide significant results shown in bold (only the smallest univariate result highlighted since this corresponds to the *P* value for the group of single phenotype analyses. Note, all univariate results are Nyholt-Šidák corrected). The difference in terms of orders of magnitude of the MultiPhen *P* value and the smallest univariate *P* value for each SNP is given in the final column.
(PDF)
