# Supplementary figures and images for: MultiPhen: Joint Model of Multiple Phenotypes Can Increase Discovery in GWAS
Source: PLoS One. 2012 May 2;7(5):e34861. doi: 10.1371/journal.pone.0034861 (PMC3342314; doi:10.1371/journal.pone.0034861)

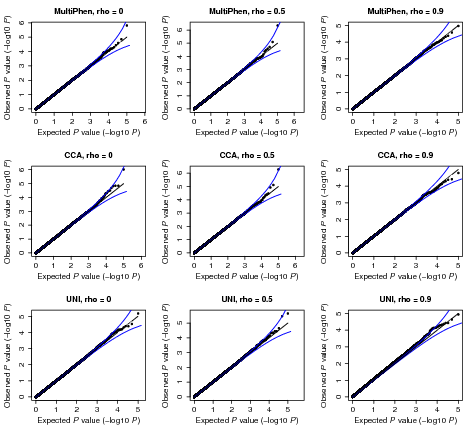

Supplement: Figure S1 — Behaviour under the null: without outliers, MAF = 30%. QQ-plots, corresponding to MultiPhen, CCA and the univariate approach (Nyolt-Šidák corrected) applied to data simulated under the null hypothesis of no SNP-phenotype effects (100000 replicates for each), sample size N = 5000, with MAF of simulated SNPs of 30% and 2 normally distributed phenotypes (without outliers) and correlations between the phenotypes of 0, 0.5 and 0.9. The plots show that all three methods have an appropriate null distribution, even when the correlation between the phenotypes is high. (TIFF) [file pone.0034861.s001.tif]

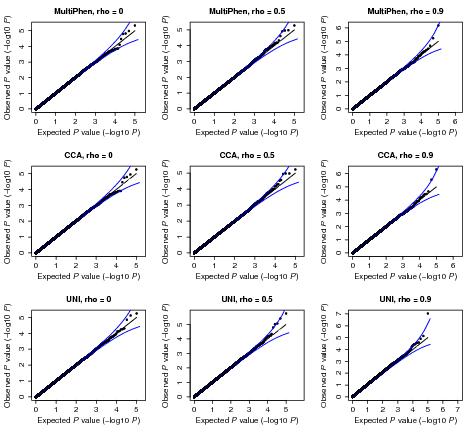

Supplement: Figure S2 — Behaviour under the null: without outliers, MAF = 0.5%. QQ-plots, corresponding to MultiPhen, CCA and the univariate approach (Nyolt-Šidák corrected) applied to data simulated under the null hypothesis of no SNP-phenotype effects (100000 replicates for each), sample size N = 5000, with MAF of simulated SNPs of 0.5% and 2 normally distributed phenotypes (without outliers) and correlations between the phenotypes of 0, 0.5 and 0.9. The plots show that all three methods have an appropriate null distribution, even when the correlation between the phenotypes is high. (TIFF) [file pone.0034861.s002.tif]

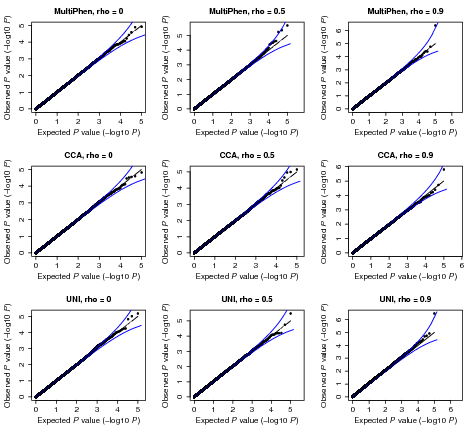

Supplement: Figure S3 — Behaviour under the null: with outliers, MAF = 30%. QQ-plots, corresponding to MultiPhen, CCA and the univariate approach (Nyolt-Šidák corrected) applied to data simulated under the null hypothesis of no SNP-phenotype effects (100000 replicates for each), sample size N = 5000, with MAF of simulated SNPs of 30% and 2 phenotypes where one has an outlier distribution and correlations between the phenotypes of 0, 0.5 and 0.9. The plots show that all three methods have an appropriate null distribution, even when the correlation between the phenotypes is high. (TIFF) [file pone.0034861.s003.tif]

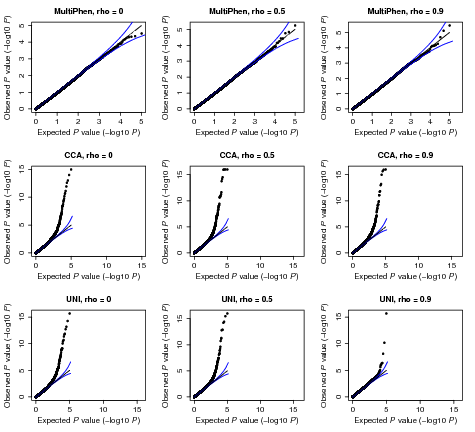

Supplement: Figure S4 — Behaviour under the null: with outliers, MAF = 0.5%. QQ-plots, corresponding to MultiPhen, CCA and the univariate approach (Nyolt-Šidák corrected) applied to data simulated under the null hypothesis of no SNP-phenotype effects (100000 replicates for each), sample size N = 5000, with MAF of simulated SNPs of 0.5% and 2 phenotypes where one has an outlier distribution and correlations between the phenotypes of 0, 0.5 and 0.9. The plots show that while MultiPhen has an appropriate null distribution, even when the correlation between the phenotypes is high, the other two approaches have a highly inflated null distribution, which would produce an extremely high type 1 error rate, irrespective of the correlation between the phenotypes. (TIFF) [file pone.0034861.s004.tif]

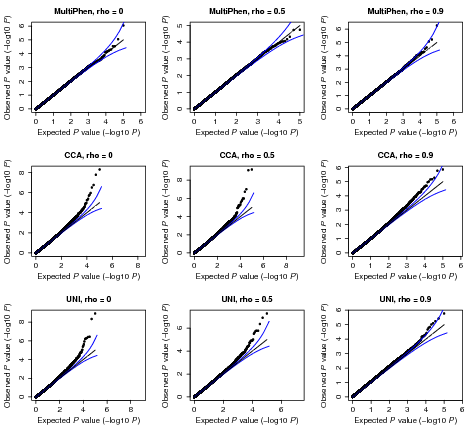

Supplement: Figure S5 — Behaviour under the null: with outliers, MAF = 5% (N = 200). QQ-plots, corresponding to MultiPhen, CCA and the univariate approach (Nyolt-Šidák corrected) applied to data simulated under the null hypothesis of no SNP-phenotype effects (100000 replicates for each), sample size N = 200, with MAF of simulated SNPs of 5% and 2 phenotypes where one has an outlier distribution and correlations between the phenotypes of 0, 0.5 and 0.9. The plots show that while MultiPhen has an appropriate null distribution, even when the correlation between the phenotypes is high, the other two approaches have an inflated null distribution. Thus CCA and the univariate approach can have a high type 1 error rate for common, as well as rare, variants. (TIFF) [file pone.0034861.s005.tif]

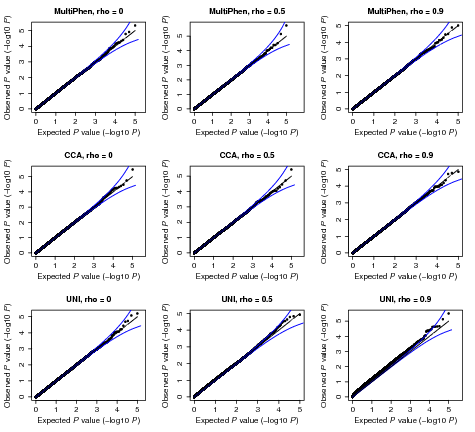

Supplement: Figure S6 — Behaviour under the null: binary phenotypes, MAF = 30%. QQ-plots, corresponding to MultiPhen, CCA and the univariate approach (Nyolt-Šidák corrected) applied to case-control study data simulated under the null hypothesis of no SNP-phenotype effects (100000 replicates for each), with sample size N = 5000 such that the first phenotype has 50% cases and controls whereas the second phenotype has 10% cases and 90% controls (case-control status defined according to the simulated values of underlying normally distributed continuous phenotypes). The MAF of the simulated SNPs is 30% and the correlations between the phenotypes (on a liability scale) are 0, 0.5 and 0.9. The plots show that all three methods have an appropriate null distribution when there are no SNP-phenotype effects, even when correlation between the phenotypes is high. (TIFF) [file pone.0034861.s006.tif]

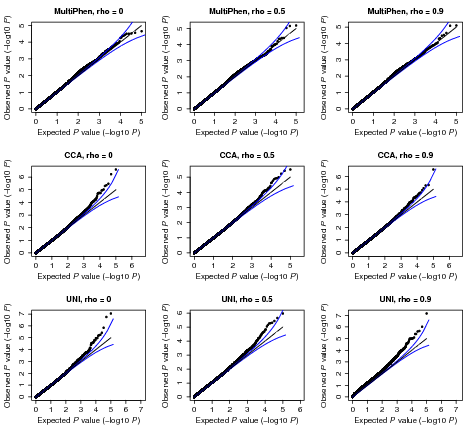

Supplement: Figure S7 — Behaviour under the null: binary phenotypes, MAF = 0.5%. QQ-plots, corresponding to MultiPhen, CCA and the univariate approach (Nyolt-Šidák corrected) applied to case-control study data simulated under the null hypothesis of no SNP-phenotype effects (100000 replicates for each), with sample size N = 5000 such that the first phenotype has 50% cases and controls whereas the second phenotype has 10% cases and 90% controls (case-control status defined according to the simulated values of underlying normally distributed continuous phenotypes). The MAF of the simulated SNPs is 0.5% and the correlations between the phenotypes (on a liability scale) are 0, 0.5 and 0.9. The plots show that while MultiPhen has an appropriate null distribution, even when the correlation between the phenotypes is high, the other two approaches have an inflated null distribution. (TIFF) [file pone.0034861.s007.tif]

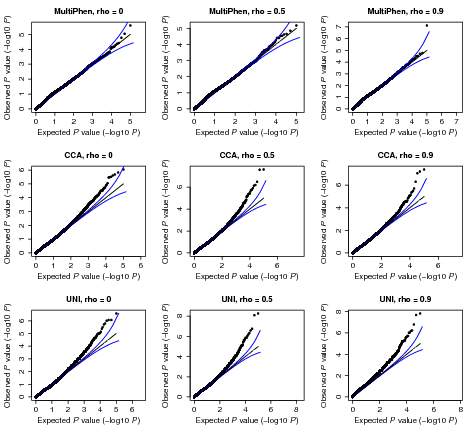

Supplement: Figure S8 — Behaviour under the null: binary phenotypes, MAF = 5% (N = 200). QQ-plots, corresponding to MultiPhen, CCA and the univariate approach (Nyolt-Šidák corrected) applied to case-control study data simulated under the null hypothesis of no SNP-phenotype effects (100000 replicates for each), with sample size N = 200 such that the first phenotype has 50% cases and controls whereas the second phenotype has 10% cases and 90% controls (case-control status defined according to the simulated values of underlying normally distributed continuous phenotypes). The MAF of the simulated SNPs is 5% and the correlations between the phenotypes (on a liability scale) are 0, 0.5 and 0.9. The plots show that while MultiPhen has an appropriate null distribution, even when the correlation between the phenotypes is high, the other two approaches have an inflated null distribution. (TIFF) [file pone.0034861.s008.tif]

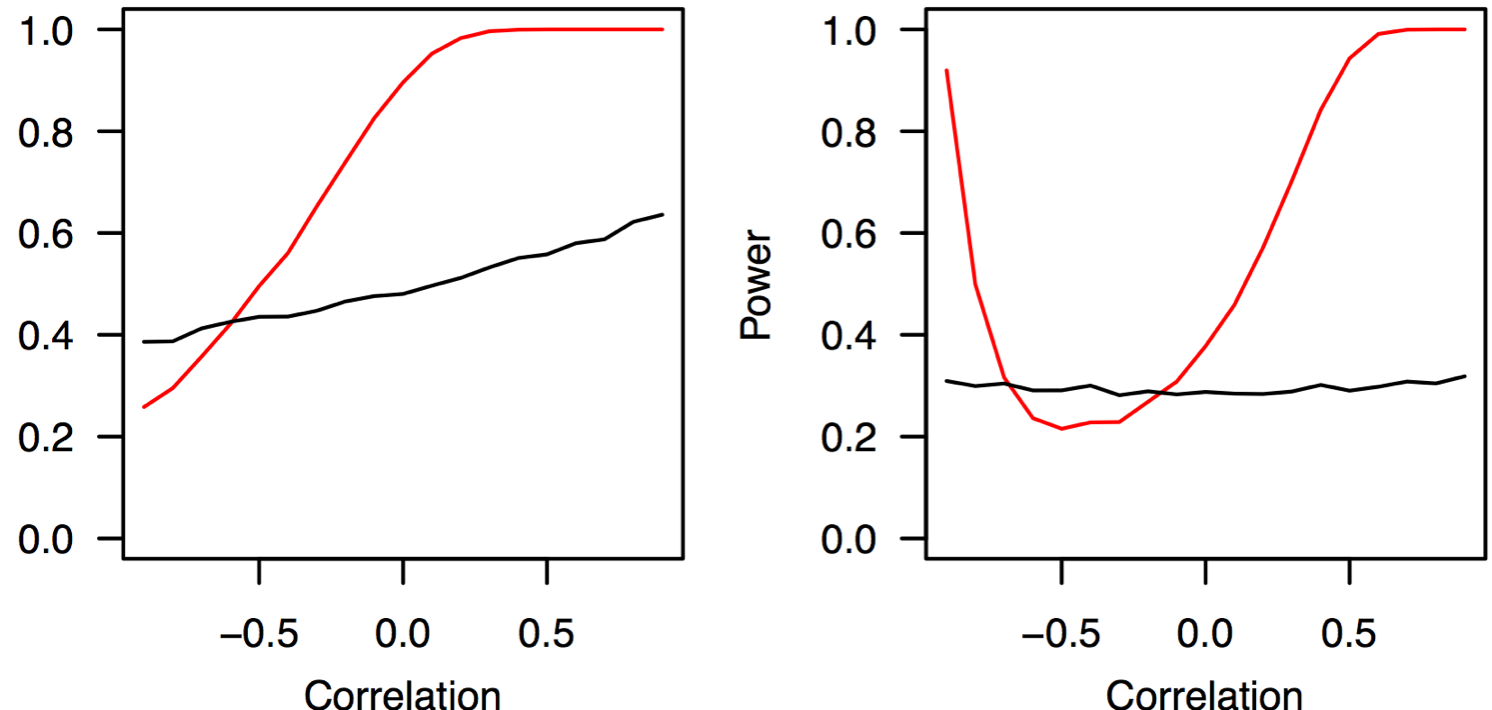

Supplement: Figure S9 — The power of MultiPhen in scenarios where the effect on the phenotypes is in the opposite direction. Power results based on simulations described in the text for MultiPhen (red lines) and the standard single-phenotype approach (black lines). Left panel: causal variant explains 0.5% of phenotypic variance of both phenotypes but in opposite directions. Right panel: causal variant explains 0.5% of the phenotypic variance of the first phenotype and 0.1% of the variance in the second phenotype in the opposite direction of the effect on the first. The X-axis corresponds to the correlation (Pearson’s correlation coefficient, r) between the phenotypes. (TIFF) [file pone.0034861.s009.tif]

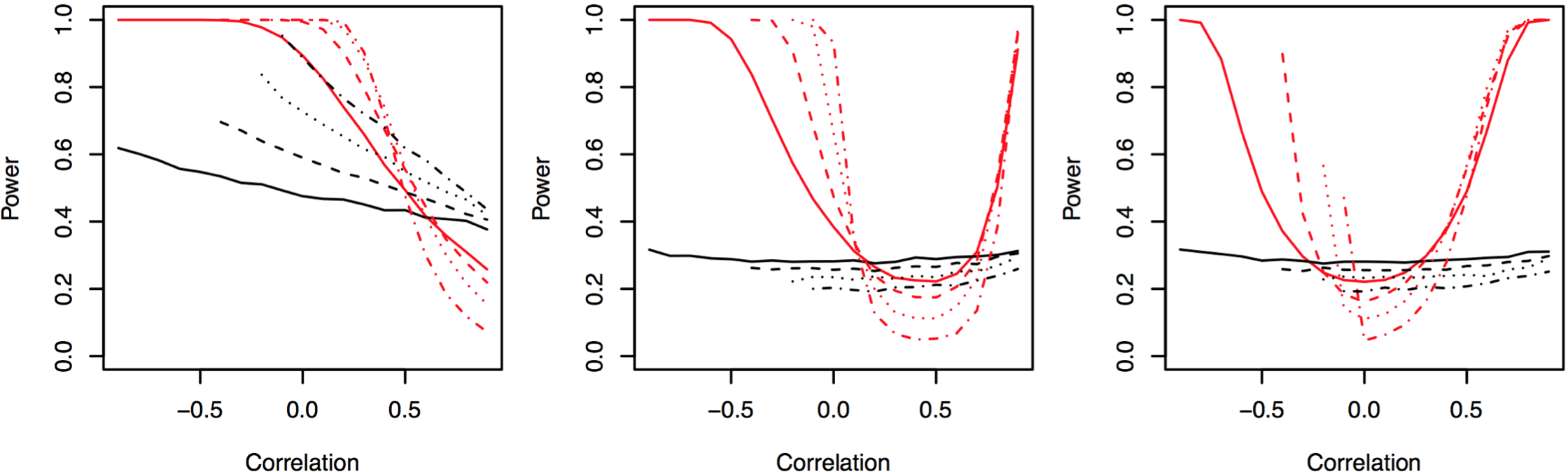

Supplement: Figure S10 — The power of MultiPhen applied to two, three, five and ten phenotypes. Power results based on simulations described in the text for MultiPhen (red lines) and the standard single-phenotype approach (black lines), for 2 (bold), 3 (dashed), 5 (dotted), and 10 (dashed/dotted) phenotypes. Left panel: causal variant explains 0.5% of phenotypic variance of each phenotype. Middle panel: causal variant explains 0.5% of the phenotypic variance of the first phenotype and 0.1% of the variance in each of the other phenotypes. Right panel: causal variant explains 0.5% of phenotypic variance of the first phenotype and 0% of each of the other phenotypes. The X-axis corresponds to the correlation (Pearson’s correlation coefficient, r) between all pairs of phenotypes (that is, the correlation between each pair of phenotypes is the same at each point of the X-axis). The results are truncated when the pairwise correlations are low for multiple phenotypes because the corresponding correlation matrices of the phenotypes are not positive definite and so the multivariate gaussian distributions cannot be sampled via the Cholesky decomposition that we perform. (TIFF) [file pone.0034861.s010.tif]

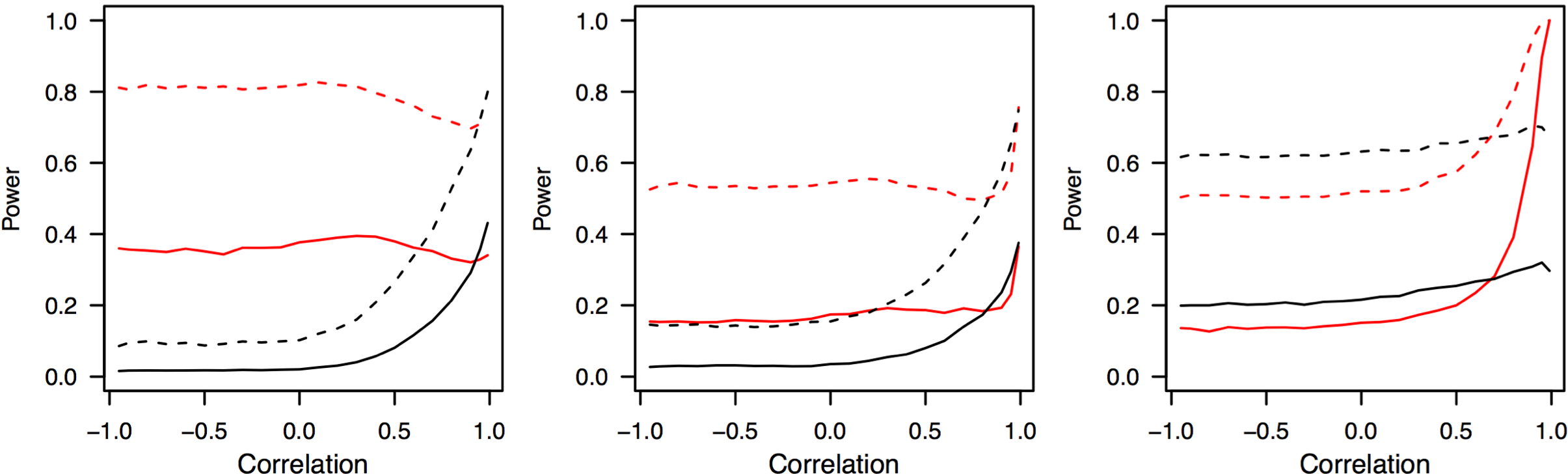

Supplement: Figure S11 — The power of MultiPhen applied to two case-control phenotypes. Power results based on simulations described in the text for MultiPhen (red lines) and the standard single-phenotype approach (black lines) applied to two independent case-control studies, each with 1000 cases and 1000 controls where case-control status is used as the predictor variables. Heritability on the liability scale is 0.1% for one phenotype and 0.1% (left panel), 0.05% (middle panel) and 0% (right panel) for the other phenotype. Solid and dashed are for disease prevalences of 1% and 0.5%, respectively, for both studies. Correlation on the X-axis is with respect to the liability scale. (TIFF) [file pone.0034861.s011.tif]

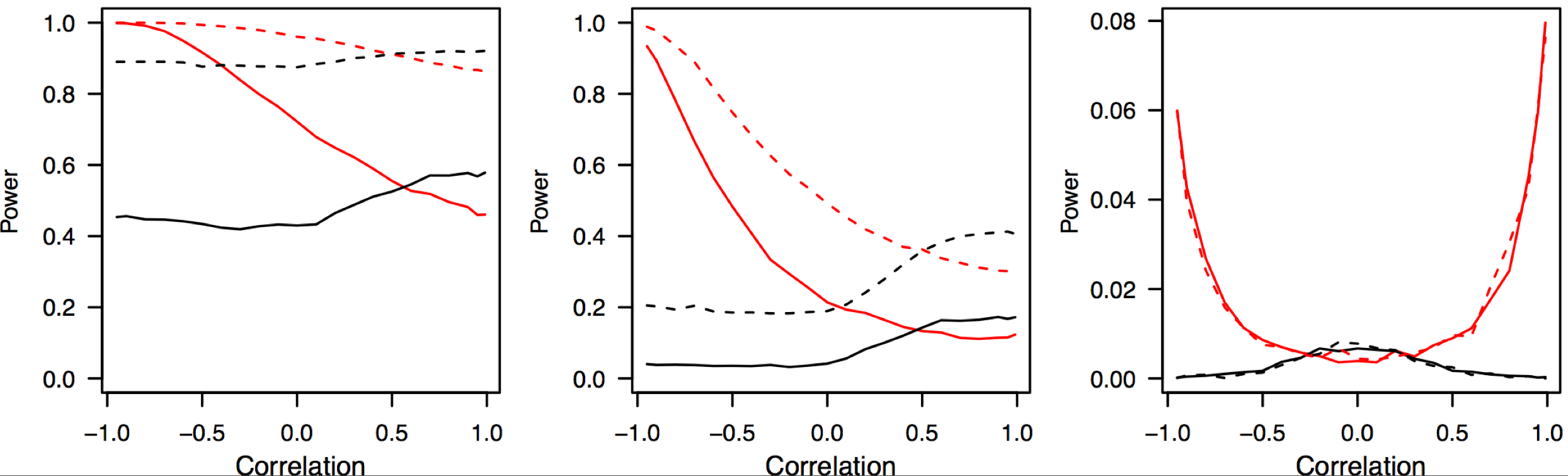

Supplement: Figure S12 — The power of MultiPhen applied to one case-control and one quantitative phenotype. The power of MultiPhen applied to a case-control study with 1000 cases and 1000 controls using case-control status and a single measured quantitative phenotype as predictor variables. Heritability of the continuous phenotype is 0.2%; heritability of the binary trait on the liability scale is: 0.5% (a), 0.1% (b) and 0% (c). Solid and dashed lines are for disease prevalence of 1% and 0.5% respectively. Correlation on the X-axis is with respect to the liability scale. (TIFF) [file pone.0034861.s012.tif]

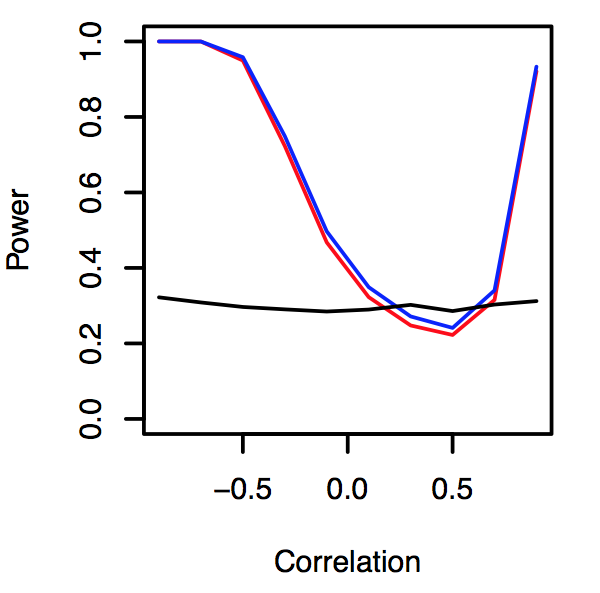

Supplement: Figure S13 — The power of CCA, compared to MultiPhen and the univariate approach. Power results based on simulations described in the text for CCA (blue), MultiPhen (red) and the single-phenotype approach (black), when applied to two continuous normally distributed phenotypes where the causal variant explains 0.5% of the phenotypic variance of the first phenotype and 0.1% of the variance of the other phenotype, with the simulated SNPs having MAF = 30%. The X-axis corresponds to the correlation (Pearson’s correlation coefficient, r) between the phenotypes. The difference in statistical power between CCA and MultiPhen in this scenario is reflective of the difference in power when the effect of the causal variants on the phenotypes differs and for different allele frequencies of the causal variant (data not shown). (TIFF) [file pone.0034861.s013.tif]
