# Supplementary material for: MultiPhen: Joint Model of Multiple Phenotypes Can Increase Discovery in GWAS
Source: PLoS One. 2012 May 2;7(5):e34861. doi: 10.1371/journal.pone.0034861 (PMC3342314; doi:10.1371/journal.pone.0034861)
Supplement: Table S1 — Behaviour under the null: r = 0. The table relates to the simulation study to test the behaviour of the different methods under the null hypothesis of no association, described in the text and presented in Figures S1–S8. The elements of the table correspond to the number of results with P values smaller than that of the corresponding column header (expected number given in the second row of the column header), with the simulation scenario given in the left-hand column. This table gives the results for all simulations where the two phenotypes have a correlation of r = 0. (PDF) [file pone.0034861.s014.pdf]

Behaviour under the null: rho=0

**Scenario**	**P-value**	**0.05**	**0.01**	**1e-3**	**1e-4**	**1e-5**	**1e-6**	**1e-7**	**1e-8**
	**E(N)<P**	**5000**	**1000**	**100**	**10**	**1**	**0.1**	**0.01**	**1e-3**
continuous	MultiPhen	5056	1037	99	12	1	0		
without outliers	CCA	5023	1044	102	15	1	1	0	
MAF=30%	UNI	4993	1025	115	13	1	0		
continuous	MultiPhen	5069	1052	96	6	1	0		
without outliers	CCA	5075	1031	96	5	1	0		
MAF=0.5%	UNI	5110	1029	104	9	2	0		
continuous	MultiPhen	5219	1085	128	16	0			
without outliers	CCA	4928	1009	105	13	0			
MAF=5%, N=200	UNI	4869	993	102	8	0			
continuous	MultiPhen	5095	1042	105	7	0			
with outliers	CCA	5079	1006	113	9	0			
MAF=30%	UNI	5076	1005	121	11	2	0		
continuous	MultiPhen	4461	918	89	8	0			
with outliers	CCA	5191	1345	308	101	60	35	24	19
MAF=0.5%	UNI	5326	1456	342	131	70	40	28	20
continuous	MultiPhen	5181	1091	115	11	2	1	0	
with outliers	CCA	5342	1300	205	33	10	4	2	1
MAF=5%, N=200	UNI	5443	1349	211	40	12	7	2	2
binary	MultiPhen	5090	1017	100	11	1	0		
MAF=30%	CCA	5104	989	93	13	1	0		
	UNI	5066	1015	95	14	2	0		
binary	MultiPhen	5339	1319	124	7	1	0		
MAF=0.5%	CCA	4881	1100	163	29	5	2	0	
	UNI	4771	1079	155	34	8	2	1	0
binary	MultiPhen	5925	1038	105	11	2	0		
MAF=5%, N=200	CCA	4875	1176	203	42	8	1	0	
	UNI	4781	1199	230	49	11	4	0	
