# Supplementary material for: MultiPhen: Joint Model of Multiple Phenotypes Can Increase Discovery in GWAS
Source: PLoS One. 2012 May 2;7(5):e34861. doi: 10.1371/journal.pone.0034861 (PMC3342314; doi:10.1371/journal.pone.0034861)
Supplement: Table S2 — Behaviour under the null: r = 0.5. The table relates to the simulation study to test the behaviour of the different methods under the null hypothesis of no association, described in the text and presented in Figures S1–S8. The elements of the table correspond to the number of results with P values smaller than that of the corresponding column header (expected number given in the second row of the column header), with the simulation scenario given in the left-hand column. This table gives the results for all simulations where the two phenotypes have a correlation of r = 0.5. (PDF) [file pone.0034861.s015.pdf]

Behaviour under the null: rho=0.5

**Scenario**	**P-value**	**0.05**	**0.01**	**1e-3**	**1e-4**	**1e-5**	**1e-6**	**1e-7**	**1e-8**
	**E(N)<P**	**5000**	**1000**	**100**	**10**	**1**	**0.1**	**0.01**	**1e-3**
continuous	MultiPhen	4997	962	106	8	2	1	0	
without outliers	CCA	4951	970	96	8	2	1	0	
MAF=30%	UNI	5203	1031	115	12	2	0		
continuous	MultiPhen	5030	977	112	12	1	0		
without outliers	CCA	4993	973	112	12	1	0		
MAF=0.5%	UNI	5345	1092	103	13	4	0		
continuous	MultiPhen	5268	1079	127	15	1	0		
without outliers	CCA	5042	1010	105	10	1	0		
MAF=5%, N=200	UNI	5287	1126	117	11	0			
continuous	MultiPhen	5041	988	102	14	3	0		
with outliers	CCA	4995	977	103	8	1	0		
MAF=30%	UNI	5350	1042	104	11	1	0		
continuous	MultiPhen	4513	952	83	8	1	0		
with outliers	CCA	5244	1419	317	130	74	51	41	26
MAF=0.5%	UNI	5612	1446	293	107	57	37	26	19
continuous	MultiPhen	5220	1111	108	8	0			
with outliers	CCA	5336	1282	196	31	16	4	3	2
MAF=5%, N=200	UNI	5562	1316	174	30	9	3	1	0
binary	MultiPhen	4866	940	93	11	2	0		
MAF=30%	CCA	4959	955	98	9	1	0		
	UNI	5554	1047	111	13	0			
binary	MultiPhen	5485	1315	107	10	2	0		
MAF=0.5%	CCA	4942	1048	152	24	6	2	1	0
	UNI	5455	1231	177	31	8	2	1	0
binary	MultiPhen	5759	989	94	14	1	0		
MAF=5%, N=200	CCA	4608	1087	204	50	17	5	2	0
	UNI	5056	1383	266	61	22	7	2	2
