# Supplementary material for: MultiPhen: Joint Model of Multiple Phenotypes Can Increase Discovery in GWAS
Source: PLoS One. 2012 May 2;7(5):e34861. doi: 10.1371/journal.pone.0034861 (PMC3342314; doi:10.1371/journal.pone.0034861)
Supplement: Table S3 — Behaviour under the null: r = 0.9. The table relates to the simulation study to test the behaviour of the different methods under the null hypothesis of no association, described in the text and presented in Figures S1–S8. The elements of the table correspond to the number of results with P values smaller than that of the corresponding column header (expected number given in the second row of the column header), with the simulation scenario given in the left-hand column. This table gives the results for all simulations where the two phenotypes have a correlation of r = 0.9. (PDF) [file pone.0034861.s016.pdf]

Behaviour under the null: rho=0.9

**Scenario**	**P-value**<br>**E(N)<P**	**0.05**<br>**5000**	**0.01**<br>**1000**	**1e-3**<br>**100**	**1e-4**<br>**10**	**1e-5**<br>**1**	**1e-6**<br>**0.1**	**1e-7**<br>**0.01**	**1e-8**<br>**1e-3**
continuous	MultiPhen	5046	1017	112	8	0			
without outliers	CCA	5019	1002	96	8	0			
MAF=30%	UNI	6044	1278	134	19	0			
continuous	MultiPhen	4996	1008	92	10	2	1	0	
without outliers	CCA	4984	1003	87	10	2	1	0	
MAF=0.5%	UNI	6009	1238	120	14	2	1	1	0
continuous	MultiPhen	5299	1130	115	16	1	0		
without outliers	CCA	5080	1020	99	13	1	0		
MAF=5%, N=200	UNI	6017	1282	135	15	1	0		
continuous	MultiPhen	4892	963	94	9	1	1	0	
with outliers	CCA	4870	929	89	6	1	0		
MAF=30%	UNI	5904	1176	126	15	1	1	0	
continuous	MultiPhen	4473	912	101	9	2	0		
with outliers	CCA	5187	1393	351	124	64	37	27	21
MAF=0.5%	UNI	5974	1277	170	21	9	6	3	3
continuous	MultiPhen	5264	1063	108	17	3	1	0	
with outliers	CCA	5432	1236	191	30	6	0		
MAF=5%, N=200	UNI	5989	1298	129	17	5	0		
binary	MultiPhen	4921	971	94	9	1	0		
MAF=30%	CCA	4908	967	106	7	0			
	UNI	7844	1585	157	18	2	0		
binary	MultiPhen	5346	1351	134	11	1	0		
MAF=0.5%	CCA	4821	1079	166	28	2	0		
	UNI	7692	1675	256	49	10	0		
binary	MultiPhen	5814	1029	110	14	1	1	1	0
MAF=5%, N=200	CCA	4889	1100	179	43	11	4	3	0
	UNI	7287	1890	341	66	24	7	2	0
