# Supplementary material for: MultiPhen: Joint Model of Multiple Phenotypes Can Increase Discovery in GWAS
Source: PLoS One. 2012 May 2;7(5):e34861. doi: 10.1371/journal.pone.0034861 (PMC3342314; doi:10.1371/journal.pone.0034861)
Supplement: Table S4 — Correlation matrix for the 4 lipids (CHOL, TRIG, HDL, LDL) based on the NFBC1966 data. The upper triangular elements of the correlation matrix show the pairwise Pearson’s correlation coefficient (r) between each pair of the traits: total cholesterol (CHOL), triglycerides (TRIG), high-density lipoprotein (HDL) and low-density lipoprotein (LDL). (PDF) [file pone.0034861.s017.pdf]

Correlation matrix for the 4 lipids (CHOL, TRIG, HDL, LDL) based on the NFBC1966 data

	CHOL	TRIG	HDL	LDL
CHOL	1	0.16	0.89	0.43
TRIG	-	1	-0.12	-0.27
HDL	-	-	1	0.28
LDL	-	-	-	1
