# Supplementary material for: MultiPhen: Joint Model of Multiple Phenotypes Can Increase Discovery in GWAS
Source: PLoS One. 2012 May 2;7(5):e34861. doi: 10.1371/journal.pone.0034861 (PMC3342314; doi:10.1371/journal.pone.0034861)
Supplement: Table S5 — Results under standard GWAS and MultiPhen approaches for genome-wide significant SNPs: CHOL-TRIG-HDL-LDL combination. Results compare univariate and MultiPhen P values, presented on the -log10 scale for ease of comparison, for all SNPs with genome-wide significant P values (>7.301 on the -log10 scale) from either approach. Genome-wide significant results shown in bold (only the smallest univariate result highlighted since this corresponds to the P value for the group of single phenotype analyses. Note, all univariate results are Nyholt-Šidák corrected). The difference in terms of orders of magnitude of the MultiPhen P value and the smallest univariate P value for each SNP is given in the final column. (PDF) [file pone.0034861.s018.pdf]

Results under standard GWAS and MultiPhen approaches for genome-wide significant SNPs: CHOL-TRIG-HDL-LDL combination

SNPs	CHOL	TRIG	HDL	LDL	MultiPhen	Order diff
rs3764261	0.47	1.22	**25.59**	0.38	**22.24**	-3.35
rs629301	8.12	-0.33	0.26	**12.18**	**10.80**	-1.38
rs1042034	4.51	5.01	4.47	6.66	**9.60**	2.94
rs1532085	1.50	0.34	**8.80**	-0.37	**9.34**	0.54
rs4420638	8.65	0.83	1.21	**12.66**	**8.92**	-3.74
rs174546	3.26	2.69	0.66	4.86	**8.52**	3.66
rs964184	2.31	**10.71**	2.55	1.21	**8.05**	-2.66
rs12678919	-0.41	6.24	3.81	-0.35	**7.75**	1.51
rs1367117	6.73	-0.03	0.57	**9.24**	**7.34**	-1.90
rs6511720	6.17	0.38	-0.04	**8.36**	5.61	-2.75
rs1260326	0.94	**7.79**	0.36	0.21	5.44	-2.35
