# Supplementary material for: MultiPhen: Joint Model of Multiple Phenotypes Can Increase Discovery in GWAS
Source: PLoS One. 2012 May 2;7(5):e34861. doi: 10.1371/journal.pone.0034861 (PMC3342314; doi:10.1371/journal.pone.0034861)
Supplement: Table S6 — Results under standard GWAS and MultiPhen approaches for genome-wide significant SNPs: TRIG-HDL-LDL combination. Results compare univariate and MultiPhen P values, presented on the -log10 scale for ease of comparison, for all SNPs with genome-wide significant P values (>7.301 on the -log10 scale) from either approach. Genome-wide significant results shown in bold (only the smallest univariate result highlighted since this corresponds to the P value for the group of single phenotype analyses. Note, all univariate results are Nyholt-Šidák corrected). The difference in terms of orders of magnitude of the MultiPhen P value and the smallest univariate P value for each SNP is given in the final column. (PDF) [file pone.0034861.s019.pdf]

Results under standard GWAS and MultiPhen approaches for genome-wide significant SNPs: TRIG-HDL-LDL combination

SNPs	CHOL	TRIG	HDL	LDL	MultiPhen	Order diff
rs3764261	-	1.29	**25.66**	0.45	**23.06**	-2.60
rs629301	-	-0.26	0.33	**12.25**	**11.43**	-0.82
rs1042034	-	5.08	4.54	6.73	**10.26**	3.53
rs1532085	-	0.41	**8.87**	-0.30	**9.56**	0.69
rs4420638	-	0.90	1.28	**12.73**	**9.43**	-3.30
rs174546	-	2.76	0.73	4.93	**9.07**	4.14
rs964184	-	**10.78**	2.62	1.28	**8.31**	-2.47
rs12678919	-	6.31	3.88	-0.28	**8.26**	1.95
rs1367117	-	0.04	0.64	**9.31**	**7.66**	-1.65
rs6511720	-	0.45	0.03	**8.43**	6.14	-2.29
rs1260326	-	**7.86**	0.43	0.28	5.89	-1.97
