# Supplementary material for: MultiPhen: Joint Model of Multiple Phenotypes Can Increase Discovery in GWAS
Source: PLoS One. 2012 May 2;7(5):e34861. doi: 10.1371/journal.pone.0034861 (PMC3342314; doi:10.1371/journal.pone.0034861)
Supplement: Table S7 — Results under standard GWAS and MultiPhen approaches for genome-wide significant SNPs: CHOL-HDL-LDL combination. Results compare univariate and MultiPhen P values, presented on the -log10 scale for ease of comparison, for all SNPs with genome-wide significant P values (>7.301 on the -log10 scale) from either approach. Genome-wide significant results shown in bold (only the smallest univariate result highlighted since this corresponds to the P value for the group of single phenotype analyses. Note, all univariate results are Nyholt-Šidák corrected). The difference in terms of orders of magnitude of the MultiPhen P value and the smallest univariate P value for each SNP is given in the final column. (PDF) [file pone.0034861.s020.pdf]

Results under standard GWAS and MultiPhen approaches for genome-wide significant SNPs: CHOL-HDL-LDL combination

SNPs	CHOL	TRIG	HDL	LDL	MultiPhen	Order diff
rs3764261	0.61	-	**25.73**	0.52	**23.39**	-2.34
rs629301	8.26	-	0.40	**12.32**	**11.08**	-1.24
rs1042034	4.65	-	4.61	6.80	**9.67**	2.87
rs4420638	8.79	-	1.35	**12.80**	**9.53**	-3.27
rs1532085	1.64	-	**8.94**	-0.23	**8.13**	-0.81
rs1367117	6.87	-	0.71	**9.38**	**7.70**	-1.68
rs964184	2.45	-	2.69	1.35	**7.42**	4.73
rs6511720	6.31	-	0.10	**8.50**	6.71	-1.79
