# Supplementary material for: MultiPhen: Joint Model of Multiple Phenotypes Can Increase Discovery in GWAS
Source: PLoS One. 2012 May 2;7(5):e34861. doi: 10.1371/journal.pone.0034861 (PMC3342314; doi:10.1371/journal.pone.0034861)
Supplement: Table S8 — Results under standard GWAS and MultiPhen approaches for genome-wide significant SNPs: CHOL-TRIG-LDL combination. Results compare univariate and MultiPhen P values, presented on the -log10 scale for ease of comparison, for all SNPs with genome-wide significant P values (>7.301 on the -log10 scale) from either approach. Genome-wide significant results shown in bold (only the smallest univariate result highlighted since this corresponds to the P value for the group of single phenotype analyses. Note, all univariate results are Nyholt-Šidák corrected). The difference in terms of orders of magnitude of the MultiPhen P value and the smallest univariate P value for each SNP is given in the final column. (PDF) [file pone.0034861.s021.pdf]

Results under standard GWAS and MultiPhen approaches for genome-wide significant SNPs: CHOL-TRIG-LDL combination

| SNPs | CHOL | TRIG | HDL | LDL | MultiPhen | Order diff |
|---|---|---|---|---|---|---|
| rs3764261 | 0.64 | 1.39 | - | 0.55 | **16.01** | 14.62 |
| rs629301 | 8.29 | -0.16 | - | **12.35** | **11.45** | -0.90 |
| rs1042034 | 4.68 | 5.18 | - | 6.83 | **9.69** | 2.86 |
| rs4420638 | 8.82 | 1.00 | - | **12.83** | **9.45** | -3.38 |
| rs174546 | 3.43 | 2.86 | - | 5.03 | **9.28** | 4.25 |
| rs12678919 | -0.24 | 6.41 | - | -0.18 | **8.14** | 1.73 |
| rs964184 | 2.48 | **10.88** | - | 1.38 | **8.02** | -2.86 |
| rs1367117 | 6.90 | 0.14 | - | **9.41** | **7.44** | -1.97 |
| rs6511720 | 6.34 | 0.55 | - | **8.53** | 6.34 | -2.19 |
| rs1260326 | 1.11 | **7.96** | - | 0.38 | 6.07 | -1.89 |
