# Supplementary material for: MultiPhen: Joint Model of Multiple Phenotypes Can Increase Discovery in GWAS
Source: PLoS One. 2012 May 2;7(5):e34861. doi: 10.1371/journal.pone.0034861 (PMC3342314; doi:10.1371/journal.pone.0034861)
Supplement: Table S9 — Results under standard GWAS and MultiPhen approaches for genome-wide significant SNPs: CHOL-TRIG-HDL combination. Results compare univariate and MultiPhen P values, presented on the -log10 scale for ease of comparison, for all SNPs with genome-wide significant P values (>7.301 on the -log10 scale) from either approach. Genome-wide significant results shown in bold (only the smallest univariate result highlighted since this corresponds to the P value for the group of single phenotype analyses. Note, all univariate results are Nyholt-Šidák corrected). The difference in terms of orders of magnitude of the MultiPhen P value and the smallest univariate P value for each SNP is given in the final column. (PDF) [file pone.0034861.s022.pdf]

Results under standard GWAS and MultiPhen approaches for genome-wide significant SNPs: CHOL-TRIG-HDL combination

SNPs	CHOL	TRIG	HDL	LDL	MultiPhen	Order diff
rs3764261	0.55	1.30	**25.67**	-	**22.52**	-3.15
rs629301	**8.20**	-0.25	0.34	-	**10.68**	2.48
rs1042034	4.59	5.09	4.55	-	**9.98**	4.89
rs1532085	1.58	0.42	**8.88**	-	**9.67**	0.79
rs174546	3.34	2.77	0.74	-	**8.94**	5.60
rs964184	2.39	**10.79**	2.63	-	**8.77**	-2.02
rs12678919	-0.33	6.32	3.89	-	**8.34**	2.02
rs4420638	**8.73**	0.91	1.29	-	**8.21**	-0.52
rs1367117	6.81	0.05	0.65	-	**7.75**	0.94
rs1260326	1.02	**7.87**	0.44	-	5.75	-2.12
