# Supplementary material for: MultiPhen: Joint Model of Multiple Phenotypes Can Increase Discovery in GWAS
Source: PLoS One. 2012 May 2;7(5):e34861. doi: 10.1371/journal.pone.0034861 (PMC3342314; doi:10.1371/journal.pone.0034861)
Supplement: Table S10 — Results under standard GWAS and MultiPhen approaches for genome-wide significant SNPs: CHOL-TRIG combination. Results compare univariate and MultiPhen P values, presented on the -log10 scale for ease of comparison, for all SNPs with genome-wide significant P values (>7.301 on the -log10 scale) from either approach. Genome-wide significant results shown in bold (only the smallest univariate result highlighted since this corresponds to the P value for the group of single phenotype analyses. Note, all univariate results are Nyholt-Šidák corrected). The difference in terms of orders of magnitude of the MultiPhen P value and the smallest univariate P value for each SNP is given in the final column. (PDF) [file pone.0034861.s023.pdf]

Results under standard GWAS and MultiPhen approaches for genome-wide significant SNPs: CHOL-TRIG combination

SNPs	CHOL	TRIG	HDL	LDL	MultiPhen	Order diff
rs174546	3.53	2.96	-	-	**9.58**	6.05
rs629301	**8.39**	-0.06	-	-	**9.00**	0.61
rs964184	2.58	**10.98**	-	-	**8.70**	-2.28
rs12678919	-0.14	6.51	-	-	**8.11**	1.60
rs1260326	1.21	**8.06**	-	-	6.56	-1.50
rs4420638	**8.92**	1.10	-	-	6.21	-2.71
