# Supplementary material for: MultiPhen: Joint Model of Multiple Phenotypes Can Increase Discovery in GWAS
Source: PLoS One. 2012 May 2;7(5):e34861. doi: 10.1371/journal.pone.0034861 (PMC3342314; doi:10.1371/journal.pone.0034861)
Supplement: Table S11 — Results under standard GWAS and MultiPhen approaches for genome-wide significant SNPs: CHOL-HDL combination. Results compare univariate and MultiPhen P values, presented on the -log10 scale for ease of comparison, for all SNPs with genome-wide significant P values (>7.301 on the -log10 scale) from either approach. Genome-wide significant results shown in bold (only the smallest univariate result highlighted since this corresponds to the P value for the group of single phenotype analyses. Note, all univariate results are Nyholt-Šidák corrected). The difference in terms of orders of magnitude of the MultiPhen P value and the smallest univariate P value for each SNP is given in the final column. (PDF) [file pone.0034861.s024.pdf]

Results under standard GWAS and MultiPhen approaches for genome-wide significant SNPs: CHOL-HDL combination

SNPs	CHOL	TRIG	HDL	LDL	MultiPhen	Order diff
rs3764261	0.70	-	**25.82**	-	**23.74**	-2.08
rs1042034	4.74	-	4.70	-	**10.38**	5.64
rs629301	**8.35**	-	0.49	-	**9.05**	0.70
rs1532085	1.73	-	**9.03**	-	**8.83**	-0.20
rs4420638	**8.88**	-	1.44	-	**7.62**	-1.26
rs16942887	2.44	-	6.98	-	**7.45**	0.47
rs1367117	6.96	-	0.80	-	**7.32**	0.36
