# Supplementary material for: MultiPhen: Joint Model of Multiple Phenotypes Can Increase Discovery in GWAS
Source: PLoS One. 2012 May 2;7(5):e34861. doi: 10.1371/journal.pone.0034861 (PMC3342314; doi:10.1371/journal.pone.0034861)
Supplement: Table S12 — Results under standard GWAS and MultiPhen approaches for genome-wide significant SNPs: CHOL-LDL combination. Results compare univariate and MultiPhen P values, presented on the -log10 scale for ease of comparison, for all SNPs with genome-wide significant P values (>7.301 on the -log10 scale) from either approach. Genome-wide significant results shown in bold (only the smallest univariate result highlighted since this corresponds to the P value for the group of single phenotype analyses. Note, all univariate results are Nyholt-Šidák corrected). The difference in terms of orders of magnitude of the MultiPhen P value and the smallest univariate P value for each SNP is given in the final column. (PDF) [file pone.0034861.s025.pdf]

Results under standard GWAS and MultiPhen approaches for genome-wide significant SNPs: CHOL-LDL combination

Sig. SNPs	CHOL	TRIG	HDL	LDL	MultiPhen	Order diff
rs629301	8.57	-	-	**12.63**	**12.02**	-0.61
rs3764261	0.92	-	-	0.83	**10.66**	9.74
rs4420638	9.10	-	-	**13.11**	**10.16**	-2.95
rs1367117	7.18	-	-	**9.69**	**8.33**	-1.36
rs6511720	6.62	-	-	**8.81**	**7.58**	-1.23
