# Supplementary material for: MultiPhen: Joint Model of Multiple Phenotypes Can Increase Discovery in GWAS
Source: PLoS One. 2012 May 2;7(5):e34861. doi: 10.1371/journal.pone.0034861 (PMC3342314; doi:10.1371/journal.pone.0034861)
Supplement: Table S13 — Results under standard GWAS and MultiPhen approaches for genome-wide significant SNPs: TRIG-HDL combination. Results compare univariate and MultiPhen P values, presented on the -log10 scale for ease of comparison, for all SNPs with genome-wide significant P values (>7.301 on the -log10 scale) from either approach. Genome-wide significant results shown in bold (only the smallest univariate result highlighted since this corresponds to the P value for the group of single phenotype analyses. Note, all univariate results are Nyholt-Šidák corrected). The difference in terms of orders of magnitude of the MultiPhen P value and the smallest univariate P value for each SNP is given in the final column. (PDF) [file pone.0034861.s026.pdf]

Results under standard GWAS and MultiPhen approaches for genome-wide significant SNPs: TRIG-HDL combination

Sig. SNPs	CHOL	TRIG	HDL	LDL	MultiPhen	Order diff
rs3764261	-	1.46	**25.83**	-	**23.42**	-2.41
rs1532085	-	0.58	**9.04**	-	**10.42**	1.38
rs964184	-	**10.95**	2.79	-	**9.17**	-1.78
rs12678919	-	6.48	4.05	-	**8.70**	2.22
rs1260326	-	**8.03**	0.60	-	6.38	-1.65
