# Supplementary material for: MultiPhen: Joint Model of Multiple Phenotypes Can Increase Discovery in GWAS
Source: PLoS One. 2012 May 2;7(5):e34861. doi: 10.1371/journal.pone.0034861 (PMC3342314; doi:10.1371/journal.pone.0034861)
Supplement: Table S14 — Results under standard GWAS and MultiPhen approaches for genome-wide significant SNPs: TRIG-LDL combination. Results compare univariate and MultiPhen P values, presented on the -log10 scale for ease of comparison, for all SNPs with genome-wide significant P values (>7.301 on the -log10 scale) from either approach. Genome-wide significant results shown in bold (only the smallest univariate result highlighted since this corresponds to the P value for the group of single phenotype analyses. Note, all univariate results are Nyholt-Šidák corrected). The difference in terms of orders of magnitude of the MultiPhen P value and the smallest univariate P value for each SNP is given in the final column. (PDF) [file pone.0034861.s027.pdf]

Results under standard GWAS and MultiPhen approaches for genome-wide significant SNPs: TRIG-LDL combination

Sig. SNPs	CHOL	TRIG	HDL	LDL	MultiPhen	Order diff
rs629301	-	-0.08	-	**12.43**	**12.19**	-0.24
rs4420638	-	1.08	-	**12.91**	**9.73**	-3.18
rs174546	-	2.94	-	5.11	**9.67**	4.56
rs1042034	-	5.26	-	6.91	**9.04**	2.13
rs964184	-	**10.96**	-	1.46	**8.65**	-2.31
rs1367117	-	0.22	-	**9.49**	**8.12**	-1.37
rs6511720	-	0.63	-	**8.61**	6.98	-1.63
rs1260326	-	**8.04**	-	0.46	6.71	-1.33
