# Supplementary material for: MultiPhen: Joint Model of Multiple Phenotypes Can Increase Discovery in GWAS
Source: PLoS One. 2012 May 2;7(5):e34861. doi: 10.1371/journal.pone.0034861 (PMC3342314; doi:10.1371/journal.pone.0034861)
Supplement: Table S15 — Results under standard GWAS and MultiPhen approaches for genome-wide significant SNPs: HDL-LDL combination. Results compare univariate and MultiPhen P values, presented on the -log10 scale for ease of comparison, for all SNPs with genome-wide significant P values (>7.301 on the -log10 scale) from either approach. Genome-wide significant results shown in bold (only the smallest univariate result highlighted since this corresponds to the P value for the group of single phenotype analyses. Note, all univariate results are Nyholt-Šidák corrected). The difference in terms of orders of magnitude of the MultiPhen P value and the smallest univariate P value for each SNP is given in the final column. (PDF) [file pone.0034861.s028.pdf]

Results under standard GWAS and MultiPhen approaches for genome-wide significant SNPs: HDL-LDL combination

Sig. SNPs	CHOL	TRIG	HDL	LDL	MultiPhen	Order diff
rs3764261	-	-	**25.82**	0.61	**24.29**	-1.53
rs629301	-	-	0.49	**12.41**	**11.65**	-0.76
rs4420638	-	-	1.44	**12.89**	**9.89**	-3.00
rs1042034	-	-	4.70	6.89	**9.64**	2.75
rs1532085	-	-	**9.03**	-0.14	**8.49**	-0.54
rs1367117	-	-	0.80	**9.47**	**8.40**	-1.07
rs6511720	-	-	0.19	**8.59**	**7.39**	-1.20
